# Controllable Cascade Aggregation Ion Programmed Nanomachines and Simple Isolation Enable Tumor‐Derived Small Extracellular Vesicles Detection for Enhanced Breast Cancer Assessment

**DOI:** 10.1002/advs.77177

**Published:** 2026-08-14

**Authors:** Zhihao Zhang, Zijing Liu, Xiangyue Meng, Pengjun Jiang, Jie Qu, Yu Jiang, Binxu Qiu, Jie Chen, Piaopiao Chen

**Affiliations:** ^1^ Department of Laboratory Medicine Med+X Center for Manufacturing Department of General Surgery State Key Laboratory of Biotherapy Department of Medical Oncology State Key Laboratory of Respiratory Health and Multimorbidity West China Hospital Sichuan University Chengdu Sichuan China; ^2^ Breast Disease Center West China Hospital Sichuan University Chengdu Sichuan China

**Keywords:** breast cancer diagnosis and subtyping, controllable cascade aggregation, filter isolation, ion‐programmed nanomachines, tumor‐derived sEVs

## Abstract

Small extracellular vesicles (sEVs) carry biomolecules that reflect their cellular origin, making them attractive biomarkers for breast cancer assessment and human epidermal growth factor receptor 2 (HER2)‐status discrimination. However, existing methods involve lengthy isolation procedures and exhibit poor detection performance due to severe interference from excessive impurities. In this study, we constructed a dual‐target electrochemical (EC) sensing platform based on cascade aggregation effects. Combined with a facile filter‐based isolation strategy, this enables rapid and convenient dual‐marker (epithelial cell adhesion molecule (EpCAM) and HER2) analysis of breast cancer‐derived sEVs. This mechanism relies on the target‐triggered disassembly of DNA nanospheres to release Ag^+^/Hg^2+^ for EC signaling. Subsequently, colistin specifically binds to G‐quadruplexes on unreacted nanospheres and induces their aggregation, sequestering background signal sources and preventing ion leakage. This noise‐silencing strategy improved matrix tolerance and reduced background leakage under a standardized diluted‐plasma workflow, enabling reliable detection of sEVs‐associated signals in filtration‐derived clinical samples. A proof‐of‐concept clinical study involving 63 breast cancer patients and 22 nonmalignant controls successfully demonstrated the platform's capability to distinguish cancer patients from controls. Furthermore, the platform achieved a 88.9% accuracy in differentiating HER2 status within this cohort. Overall, this streamlined “filtration‐to‐detection” platform offers a promising strategy for analyzing breast cancer‐specific sEVs signals and conducting exploratory HER2‐status assessments.

## Graphical abstract

This study developed a convenient, enzyme‐free detection platform based on controllable aggregation ion nanomachines. Its favorable anti‐interference capacity and high sensitivity meet the detection requirements for clinical samples processed via a simple filtration method within 10 min. The system enables detection of breast cancer‐derived sEVs down to 30 particles/mL within 90 min. Clinical validation on a cohort of 85 samples (including 63 cancer patients and 22 healthy controls) demonstrated excellent diagnostic accuracy (area under the curve (AUC)> 0.8) for breast cancer and HER2 status, with detection results consistent with clinical diagnostic information.

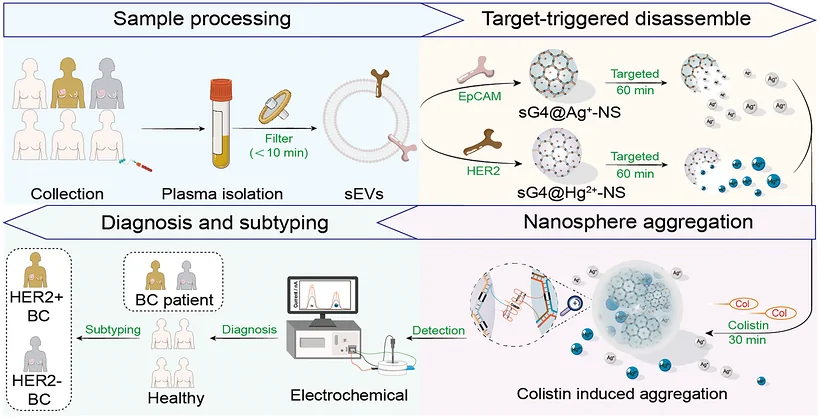

## 1 Introduction

Breast cancer remains the primary cause of cancer‐associated deaths in women globally. Early diagnosis and individualized therapy based on molecular subtyping are crucial for improving the prognosis [1, 2]. Approximately 20%–25% of breast cancer cases belong to the human epidermal growth factor receptor 2 (HER2)‐positive subtype, exhibiting a highly aggressive clinical course and inferior survival outcomes in the absence of appropriate anti‐HER2 therapy [3, 4]. Currently, HER2 status determination primarily relies on histopathological and immunohistochemical analyses of tissue specimens [5, 6]. However, these approaches are inherently invasive and unsuitable for repeated assessments or evaluating spatial heterogeneity in metastatic lesions [7, 8]. Liquid biopsy has emerged as a promising non‐invasive alternative for personalized cancer diagnosis and monitoring [9]. Among the various circulating biomarkers, small extracellular vesicles (sEVs) are more abundant in the early stages of cancer [10, 11]. Furthermore, their surface is enriched with protein biomarkers derived from parental tumor cells (for example, HER2 and epithelial cell adhesion molecule (EpCAM)), allowing cancer diagnosis and HER2 subtype discrimination through multiplex analysis [12, 13, 14, 15, 16]. Despite growing interest in sEVs as diagnostic biomarkers, cumbersome, time‐consuming isolation and detection methods have hindered their clinical application [17, 18]. Therefore, there is an urgent need to develop promising methods that enable convenient sEVs isolation and rapid multiplex detection.

Currently, the most used method for sEVs isolation is ultracentrifugation, which is time‐consuming and requires specialized equipment. Recently, alternative sEVs isolation techniques have been extensively explored to replace cumbersome ultracentrifugation. Size‐exclusion chromatography enables gentle separation while preserving sEVs integrity; however, it requires specialized equipment [19, 20]. Immunoaffinity‐based capture techniques provide high specificity but are constrained by low yield and high cost. Polymer precipitation methods are simple and equipment‐free; however, they frequently co‐isolate abundant proteins and other contaminants, compromising sEVs purity and structural integrity [21, 22]. In comparison, the membrane filtration method is simple, rapid, and requires minimal equipment. However, this method inevitably introduces substantial impurities, resulting in complex sample matrices that severely interfere with downstream detection. Although techniques, including oscillatory filtration, have improved recovery and reduced aggregation, the inherent problem of impurity interference with subsequent detection remains [23]. Consequently, an analytical method that is anti‐interference and simple is required for practical clinical applications.

Consequently, significant efforts have been devoted to developing sEVs‐detection strategies for complex biofluids. Aptamers have emerged as attractive recognition elements because of their high stability, strong affinity, and superior performance in complex matrices [24, 25, 26, 27]. However, aptamers can only recognize targets and require additional components to produce detectable signals, increasing the complexity of the detection system. The emergence of DNA nanomachines, including nanohydrogels, nanospheres, and nanonets, has enabled the integration of aptamers into nanostructures, producing integrated probes that combine recognition, signal amplification, and signal output, thereby effectively simplifying detection [28, 29, 30, 31, 32, 33]. Its large specific surface area allows the pre‐encapsulation of numerous distinct signal molecules, thereby enabling multiplexed target detection and enzyme‐free signal amplification. Nevertheless, in complex biological matrices, these nanostructures remain vulnerable to nonspecific interference and premature signal leakage, which compromises their detection performance [34]. Although surface modification strategies, including polyethylene glycol coating, have been used to improve stability, they often introduce steric hindrance, reduce target accessibility, and require complicated fabrication processes [35]. Conversely, small‐molecule‐mediated controlled aggregation provides a simple and effective alternative for stabilizing nanostructures and suppressing background interference. Notably, colistin induces compact assemblies of parallel G‐quadruplex (G4) structures *via π–π* stacking, providing a unique strategy for anti‐interference design [36].

Accordingly, we developed a simple colistin‐mediated, controllably aggregating DNA nanomachine for detecting sEVs obtained via rapid membrane filtration, thereby enabling exploratory breast cancer‐related sEVs‐associated signal analysis. Specifically, this system comprises two types of DNA nanospheres encapsulating Ag^+^ and Hg^2+^ ions, respectively, and is functionalized with aptamers targeting EpCAM and HER2. Upon specific binding to sEVs surface proteins, the nanospheres undergo target‐triggered disassembly, releasing metal ions that generate distinct electrochemical (EC) signals for multiplexed detection. Notably, colistin is introduced to selectively induce the aggregation of unreacted nanospheres through G4 interactions, thereby suppressing background ion leakage and significantly enhancing anti‐interference performance. This “signal‐on/noise‐off” strategy enables reliable detection in crude filtration‐derived samples without ultracentrifugation. Preliminary tests on limited clinical samples reveal favorable consistency between our assay results and imaging/histopathological data, demonstrating its preliminary potential for breast cancer diagnosis and molecular subtyping at the proof‐of‐concept stage.

## 2 Results and Discussion

### 2.1 Controllable Cascading Aggregated Ion‐Programmed Nanomachines

The self‐assembly of Ag^+^‐nanospheres (Ag^+^‐NS) was accomplished by conjugating Ag^+^ to the C‐rich domains at the terminal ends of Y1‐3 sequences, which enabled the C‐Ag^+^‐C coordination structure formation and therefore yielded natural palindromic sequences (Figure 1a). Similarly, Hg^2+^‐nanospheres (Hg^2+^‐NS) were assembled via T‐Hg^2+^‐T coordination by linking Hg^2+^ to T‐rich terminals of Y4‐6 sequences (Figure 1b). The detailed nucleic acid sequences are presented in Table S1. Gel electrophoresis was used to verify the successful assembly of nanospheres. To avoid complexation between metal ions in the nanospheres and thiol groups present in the loading buffer, Ag^+^ was omitted in lane 6. Meanwhile, the poly‐C segment was substituted with a palindromic sequence to promote the self‐assembly of nanospheres (Table S2). Because Y1, Y2, and Y3 can hybridize complementarily with each other, low‐molecular‐weight complexes, including Y1+Y2, Y1+Y3, and Y2+Y3, may be present in lane 5, appearing as small‐molecular‐weight bands. After annealing, new high‐molecular‐weight products appeared in lane 6, indicating the successful assembly of Y_1‐3_‐NS (Figure 1c). Y_4‐6_‐NS was characterized using the same gel electrophoresis method (Figure 1d and Table S3). Atomic force microscopy (AFM) and transmission electron microscopy (TEM) were employed to characterize the morphological features and size distribution of the synthesized nanostructures. As presented in Figure 1e1,f1, the free Y‐DNA (Y1‐3 and Y4‐6) appeared as small, uniformly dispersed dots. Conversely, following the addition of metal ions, distinct spherical nanostructures were observed for Ag^+^‐NS (Figure 1e2) and Hg^2+^‐NS (Figure 1f2). Consistent results were obtained via TEM (Figure 1g,h). To verify this finding, dynamic light scattering (DLS) and Zeta potential measurements were performed. For the Ag^+^‐NS system, the hydrodynamic diameter of the Y1‐3 mixture was approximately 10 nm, whereas the size increased to approximately 120 nm upon Ag^+^‐NS formation (Figure 1i). Zeta potential shifted from −14.5 mV (free Y1‐3) to −10.7 mV (Ag^+^‐NS) due to the neutralization of the negatively charged DNA backbone by positively charged Ag^+^ ions (+7.3 mV) (Figure 1j). Consistent trends were observed for the Hg^2+^‐NS system (Figure 1k,l). These results indicate that DNA nanospheres were successfully synthesized and are available for further analysis.

**FIGURE 1:**
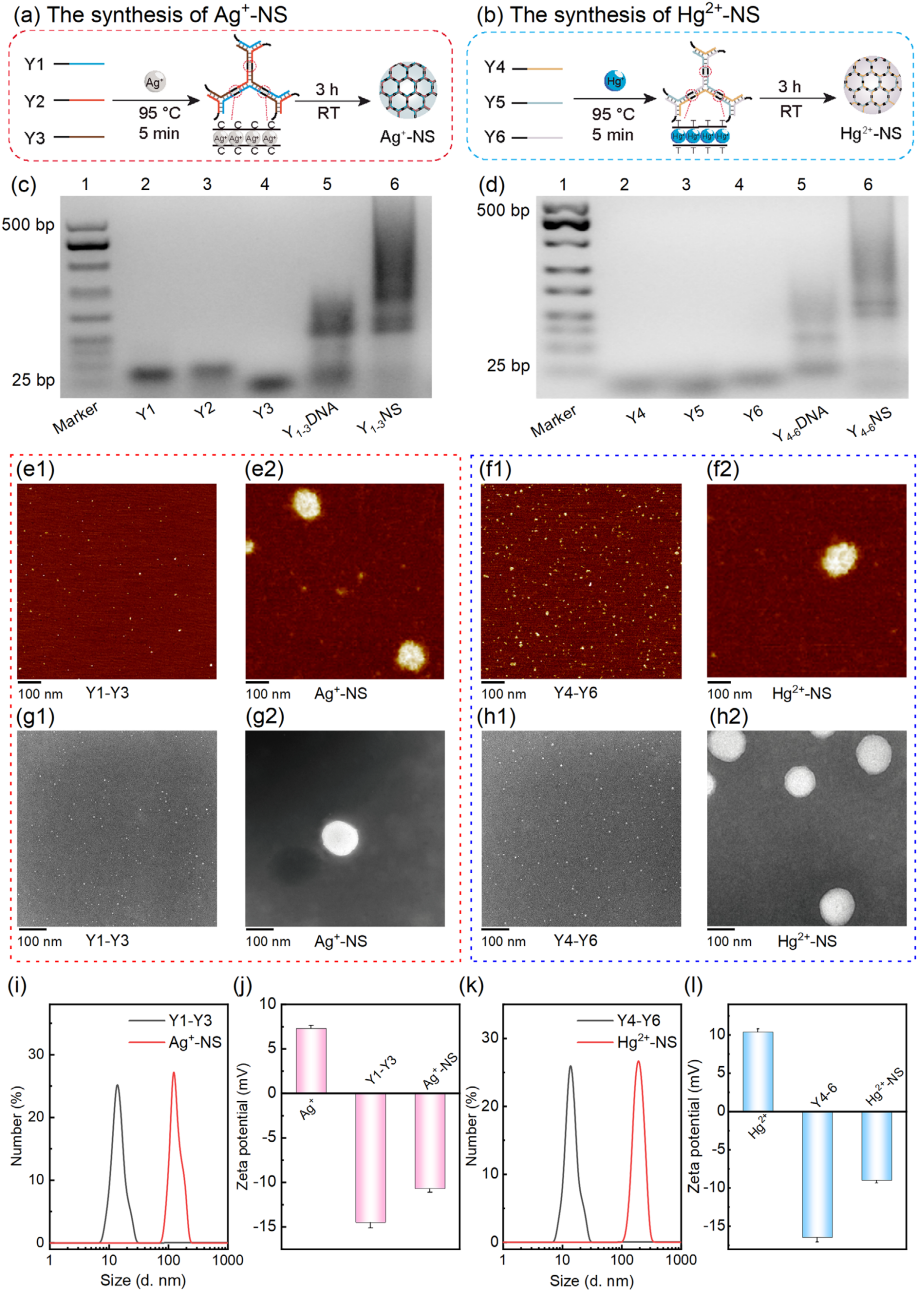
Synthesis and characterization of the DNA nanospheres. Schematic diagram of the self‐assembly synthesis of (a) Ag^+^‐NS and (b) Hg^2+^‐NS. Agarose gel electrophoresis analysis of the assembly process for (c) Ag^+^‐NS and (d) Hg^2+^‐NS. AFM (e, f) and TEM (g, h) images of Y‐DNA, metal‐NS. DLS and zeta potential analyses of the Ag^+^‐NS system (i, j) and Hg^2+^‐NS system (k, l) before and after assembly. Triple measurements calculated error bars and represented standard deviations.

To establish the basis for dual‐target detection, EC signals of Ag^+^ and Hg^2+^ were evaluated using differential pulse voltammetry (DPV). As presented in Figure 2a, the current peaks of Ag^+^ and Hg^2+^ were distinct and well‐resolved, confirming the capability for simultaneous detection without cross‐interference. To provide direct evidence for the interaction between G4 and colistin, isothermal titration calorimetry (ITC) directly confirmed the interaction between G4 and colistin, with a dissociation constant of *Kd* = 14.8 ± 9.1 µM (Figure 2b,c). The chemical structure of colistin was shown in Figure S1. In contrast, the non‐G4 single‐stranded (ssDNA) control exhibited nonspecific heat changes, further supporting the preferential binding of colistin to the G4 structure (Figure S2). This interaction was further supported by UV–vis measurements (Figure 2d), which showed an increase in apparent absorbance at 500 nm after colistin addition, consistent with aggregation‐induced turbidity. The turbidity signal was retained in the presence of Ag^+^, Hg^2+^, or plasma, whereas the protein‐containing control showed negligible absorbance at 500 nm. These results indicate that the observed aggregation was primarily driven by the specific G4‐colistin interaction, remained effective in the presence of ionic and plasma matrix components, and was not attributable to nonspecific protein adsorption (Figure S3). Subsequently, to achieve optimal aggregation efficiency, various split G4 (sG4) subtypes were screened (Tables S4–S6) [36, 37]. Figure 2e demonstrates that the signal attenuation mediated by sG4a and sG4a' was the most significant, indicating that colistin yielded the best aggregation efficiency for sG4@Ag^+^‐NS of this sG4 subtype (sG4a and sG4a'). Similarly, sG4e and sG4e' exhibited optimal aggregation‐inducing effects on Hg^2+^‐NS (Figure 2f). Circular dichroism spectroscopy confirmed that the sG4 sequences on the nanospheres could reconstitute an intact G4 structure (Figure S4). Therefore, these subtypes were selected for subsequent experiments. Control experiments confirmed that colistin and sG4 exerted non‐significant interference on EC signals of free Ag^+^ and Hg^2+^; only when the colistin concentration exceeded 500 µM did it reduce the Ag^+^ signal (Figure 2g,h). However, the concentration used in this study was well below this threshold, thereby avoiding such interference.

**FIGURE 2:**
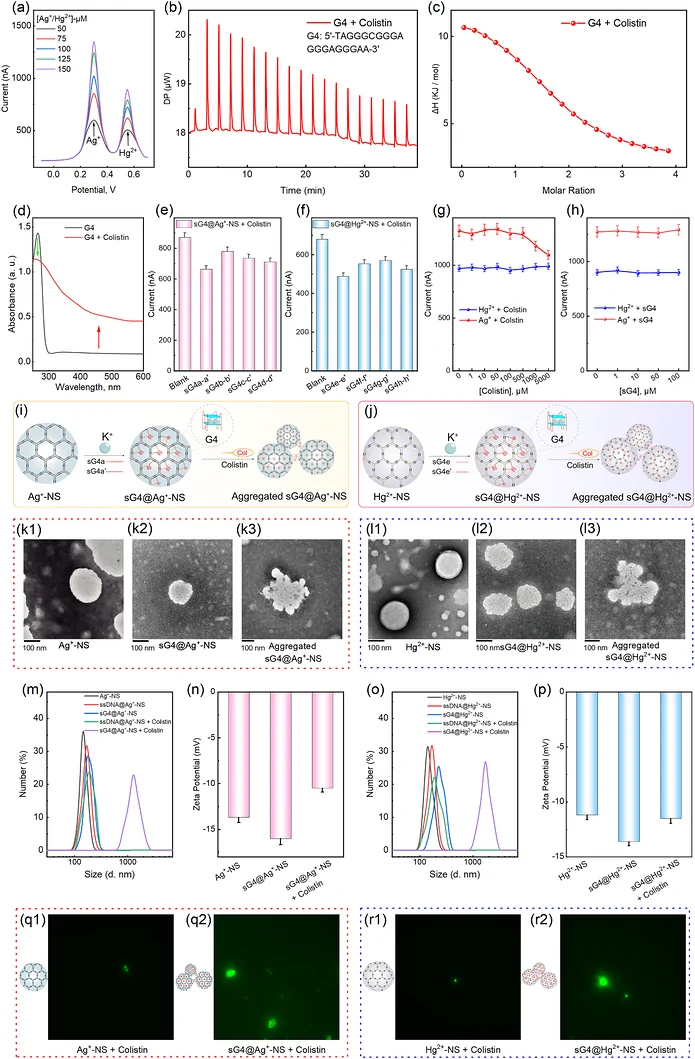
Characterization of colistin‐induced sG4@metal‐nanosphere aggregation. (a) DPV response of Ag^+^ and Hg^2+^. (b) Representative data of the titration thermograms obtained. (c) Data integration with fitted curve, using 1:1 binding model. (d) UV–vis spectra of free G4 (black) and G4 incubated with colistin (red). Colistin decreased the G4 absorbance near 260 nm and increased the 300–600 nm baseline due to aggregate‐induced light scattering. Screening of optimal sG4 subtypes for inducing aggregation of (e) Ag^+^‐NS and (f) Hg^2+^‐NS. Effects of (g) colistin and (h) split G4 on the EC signals of Ag^+^ and Hg^2+^. Schematic diagram of the aggregation of (i) Ag^+^‐NS and (j) Hg^2+^‐NS co‐promoted by colistin and split G4. (k, l) TEM images of Ag^+^‐NS, sG4@Ag^+^‐NS, aggregated sG4@Ag^+^‐NS, Hg^2+^‐NS, sG4@Hg^2+^‐NS, and aggregated sG4@Hg^2+^‐NS. DLS and Zeta potential for the Ag^+^‐NS system (m‐n) and Hg^2+^‐NS system (o, p) at different processes. Fluorescence microscopy image of (q) aggregated sG4@Ag^+^‐NS and (r) sG4@Hg^2+^‐NS stained with SYBR green I. Triple measurements calculated error bars and represented standard deviations.

The aggregation mechanism of sG4@metal‐NS, schematically illustrated in Figure 2i,j, was systematically characterized. TEM provided direct visualization of structural evolution. Ag^+^‐NS displayed a smooth and uniform spherical morphology (Figure 2k1). Upon modification with sG4, the nanosphere surface became noticeably rough (Figure 2k2), contributing to the anchoring of DNA strands. Following colistin introduction, distinct large‐scale aggregates were observed (Figure 2k3). A parallel morphological evolution was observed in the Hg^2+^‐NS system (Figure 2l), confirming the universality of this aggregation strategy. To verify these findings, DLS and Zeta potential measurements were performed. For the Ag^+^‐NS system, the hydrodynamic diameter increased from 142 nm (Ag^+^‐NS) to approximately 190 nm (sG4@Ag^+^‐NS) and finally surged to approximately 1700 nm upon colistin‐induced aggregation. In contrast, ssDNA@Ag^+^‐NS showed no appreciable increase in hydrodynamic diameter after colistin addition, further supporting the G4‐dependent nature of the aggregation process (Figure 2m). Colistin addition to sG4@Ag^+^‐NS increased the surface charge, implying that colistin promoted nanosphere aggregation (Figure 2n). Consistent results were obtained from DLS and Zeta potential analyses of the Hg^2+^‐NS system (Figure 2o,p). Moreover, fluorescence imaging with SYBR Green I staining confirmed the aggregation. In contrast to the faint, dispersed spots observed in the non‐aggregated groups (Figure 2q1,2r1), colistin‐treated sG4@metal‐NS exhibited intense, large green fluorescent clusters (Figure 2q2,r2). These results demonstrate that colistin can trigger sG4@metal‐NS aggregation, which provides a basis for improving its performance in future detection applications.

### 2.2 Performance Enhancement of the Controllably Aggregated Nanomachines

In this study, a controllably cascade‐aggregated DNA nanomachine was fabricated to improve the anti‐interference performance and stability of the sensing system. Ag^+^, Hg^2+^ (EC and mass spectrometric signals), and methylene blue (MB; with EC and fluorescent signals) were used for verification (Scheme 1). These signal molecules can be pre‐encapsulated into the DNA scaffold of the nanomachine and detected by EC workstation, fluorescence, and mass spectrometric instruments. Specifically, in the presence of the target, it binds to the aptamer embedded in the DNA nanosphere scaffold, thereby triggering the disassembly of the nanosphere and releasing the signal molecules (Ag^+^, Hg^2+^, or MB). In the absence of the target, the nanomachines remained intact, resulting in no appreciable signal generation. Based on the selective release and recognition of these signal molecules, the system produces distinct responses that enable quantitative target analyses.

**SCHEME 1:**
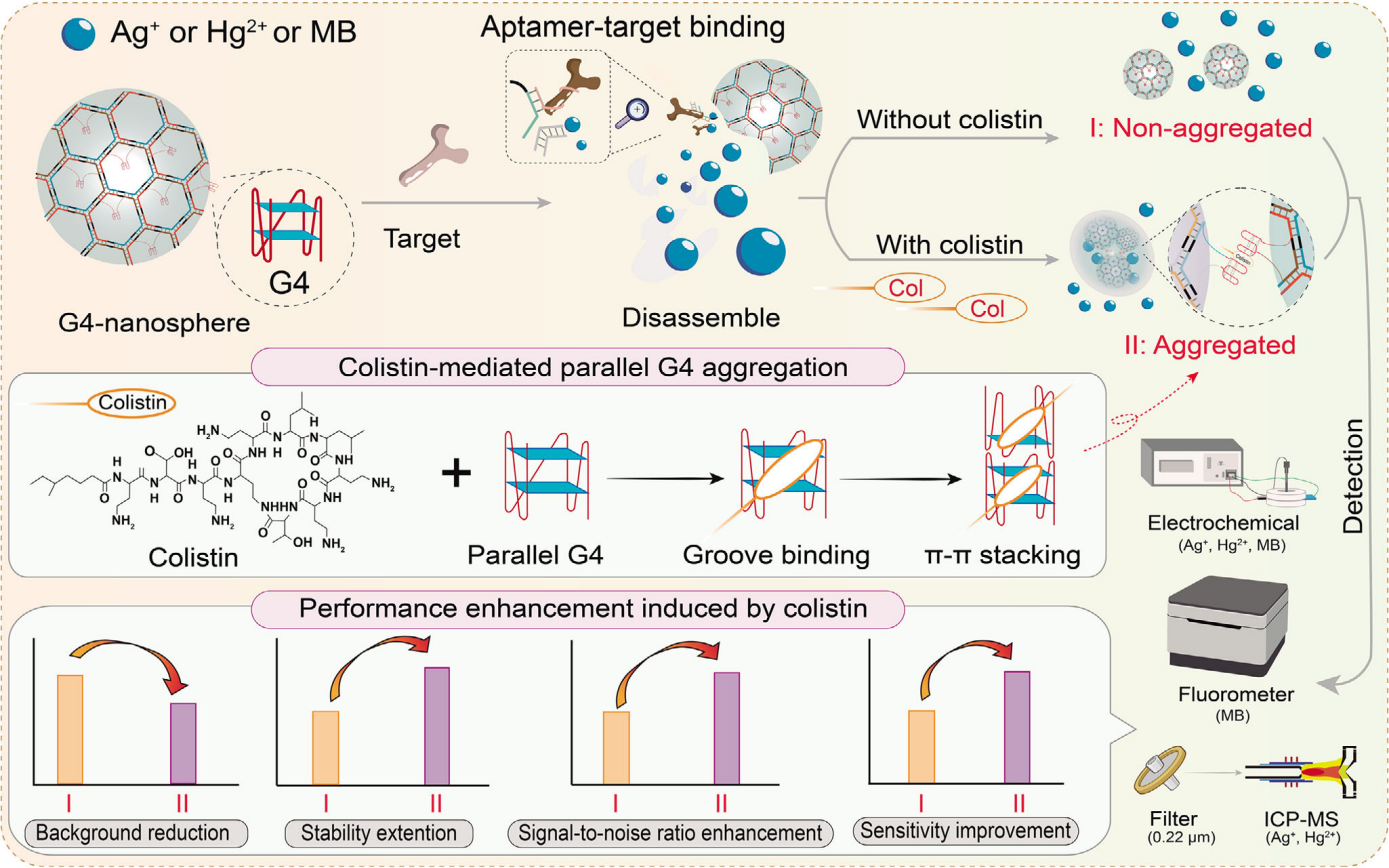
Schematic illustration of controllable cascade aggregation nanomachines.

Colistin was then introduced to enhance the anti‐interference ability and stability of the system. As a cationic polypeptide, colistin can specifically recognize and insert into the groove regions of parallel G4 through groove‐binding interactions. Moreover, the colistin‐bound G4 units can further undergo aggregation by *π–π* stacking interactions. Exploiting this mechanism, colistin acted as a supramolecular cross‐linker to induce the macroscopic aggregation of unreacted and free‐floating G4‐nanospheres. This process enhances the stability of the nanomachine and effectively suppresses nonspecific leakage, thereby reducing the background signal and significantly improving the signal‐to‐noise ratio and overall stability of the system, as presented in Figure 3.

**FIGURE 3:**
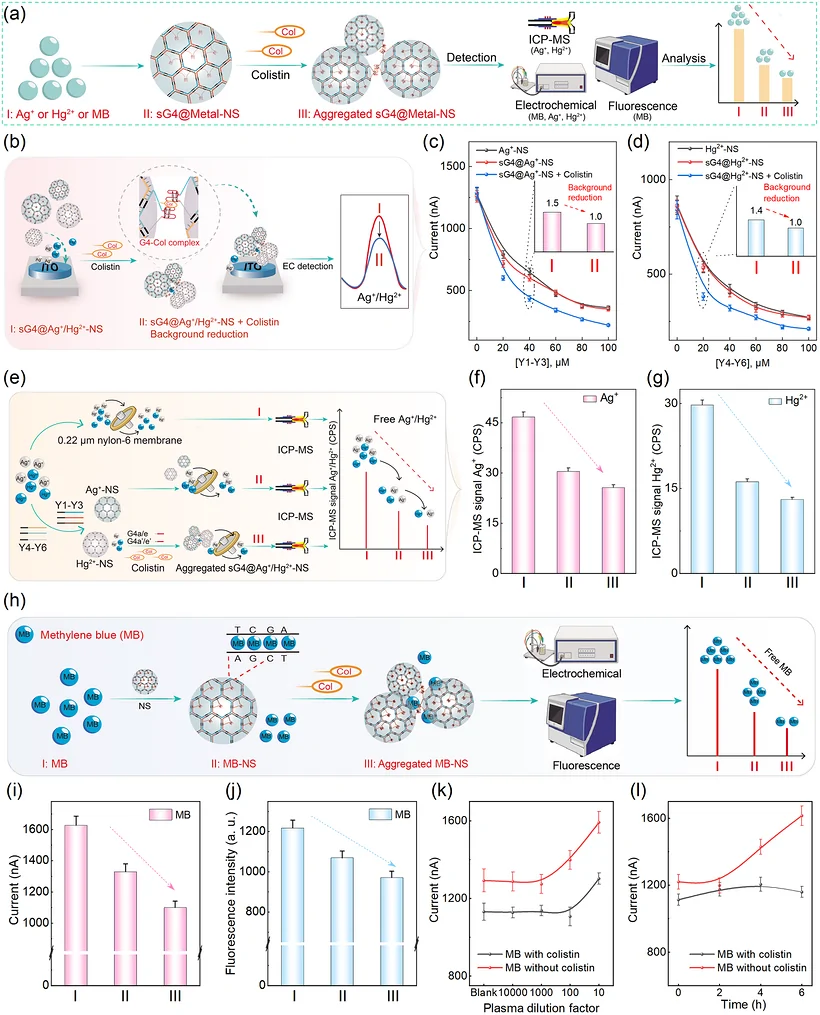
Performance of controllable, aggregated nanomachines. (a) Schematic illustration of colistin‐induced sG4@metal‐NS aggregation, leading to reduced background signals. (b) Schematic illustration of EC signals. EC responses of (c) sG4@Ag^+^‐NS and (d) sG4@Hg^2+^‐NS upon colistin addition. (e) Schematic diagram of ICP‐MS. ICP‐MS signals of (f) sG4@Ag^+^‐NS and (g) sG4@Hg^2+^‐NS upon colistin addition. (h) Schematic diagram of MB by EC and fluorescence signal. MB signals of (i) EC and (j) fluorescence upon colistin addition. Plasma stability at (k) different dilution ratios and (l) incubation time with or without colistin. Triple measurements calculated error bars and represented standard deviations.

The detection of signal molecules (Ag^+^, Hg^2+^, and MB) using multiple methods confirmed that the introduction of colistin enhanced the anti‐interference ability of the system and reduced the background signal (Figure 3a). Initially, an EC was used to validate the system (Figure 3b). As the concentrations of the DNA scaffold strands (Y1‐3 for Ag^+^ and Y4‐6 for Hg^2+^) increased, the EC currents progressively decreased, indicating the successful assembly of Ag^+^‐NS and Hg^2+^‐NS (Figure 3c,d). Notably, upon the introduction of colistin, the residual currents of sG4@Ag^+^‐NS and sG4@Hg^2+^‐NS exhibited a further significant decrease, with the background signals decreasing by approximately 50% and 40%, respectively. Similar results were observed in the inductively coupled plasma‐mass spectrometry (ICP‐MS) experiments (Figure 3e and Table S7). The reaction mixtures were filtered through a 0.22 µm nylon‐6 membrane, which retained the nanospheres and aggregates while allowing free Ag^+^ and Hg^2+^ to pass into the filtrate. As depicted in Figure 3f,g, Ag^+^ and Hg^2+^ concentrations in the filtrate of aggregated sG4@metal‐NS groups were lower than those of the free ion and non‐aggregated nanosphere. Furthermore, MB, which exhibits EC and fluorescence signals, was used to verify this phenomenon (Figure 3h) [38, 39, 40]. As presented in Figure 3i, the EC signal of MB decreased slightly upon adding DNA nanospheres, which can be attributed to the intercalation of MB into the double‐stranded DNA of the nanospheres. Upon further introduction of colistin, the electrical signal of MB decreased significantly. Consistently, the fluorescent assay of MB yielded identical results (Figure 3j). These results provide evidence that colistin‐induced aggregation significantly lowers background noise, establishing a solid foundation for enhancing the signal‐to‐noise ratio and sensitivity of the assay.

Subsequently, the effect of colistin addition on the anti‐interference capability of the sensing system in plasma was studied to evaluate its application potential in clinical sample detection. When sG4@MB‐NS was incubated with plasma at different dilution ratios, the MB signal in the colistin‐free group increased in 100‐fold diluted plasma. However, the signal in the colistin‐treated group increased significantly only at a 10‐fold dilution (Figure 3k). Furthermore, a time‐dependent stability assay was performed for both groups in 10 000‐fold diluted plasma (Figure 3l). The results exhibited that the MB signal of sG4@MB‐NS without colistin increased significantly after 4 h of incubation, whereas the signal in the colistin‐containing group remained relatively stable throughout. Collectively, these results indicate that colistin‐mediated nanosphere aggregation improves the stability and anti‐interference capacity of the sensing system under diluted‐plasma conditions.

### 2.3 sEVs Sensing Enables Assessment of Breast Cancer

These results demonstrate that the controllable aggregated nanomachine system exhibits excellent anti‐interference and detection performance. Next, using breast cancer as an example, the sEVs detection capability of this system in clinical plasma samples was verified. EpCAM (a broadly overexpressed tumor marker) and HER2 (a key therapeutic target for treatment guidance) were selected as detection targets, and an EC workstation was used as an example for signal detection (Scheme 2). Specifically, when EpCAM is present on the sEVs surface, it can bind to the EpCAM aptamer in the sG4@Ag^+^‐NS, causing their disassembly and the release of Ag^+^, which can be detected by an EC workstation [41]. Similarly, HER2‐positive sEVs triggered the disassembly of sG4@Hg^2+^‐NS by binding to the HER2 aptamer, generating an EC signal for Hg^2+^ [42]. Subsequently, colistin was introduced into the system to effectively enhance its detection performance and anti‐interference ability. In clinical samples, stronger Ag^+^ and Hg^2+^ responses indicate a higher total sEVs‐associated EpCAM or HER2, which is jointly determined by the abundance of marker‐positive sEVs and the average protein abundance per vesicle, rather than by either factor alone. Patients with breast cancer can be divided into HER2‐positive and HER2‐negative subgroups according to their HER2 expression levels, with the HER2‐positive group exhibiting a significantly higher Hg^2+^ EC signal.

**SCHEME 2:**
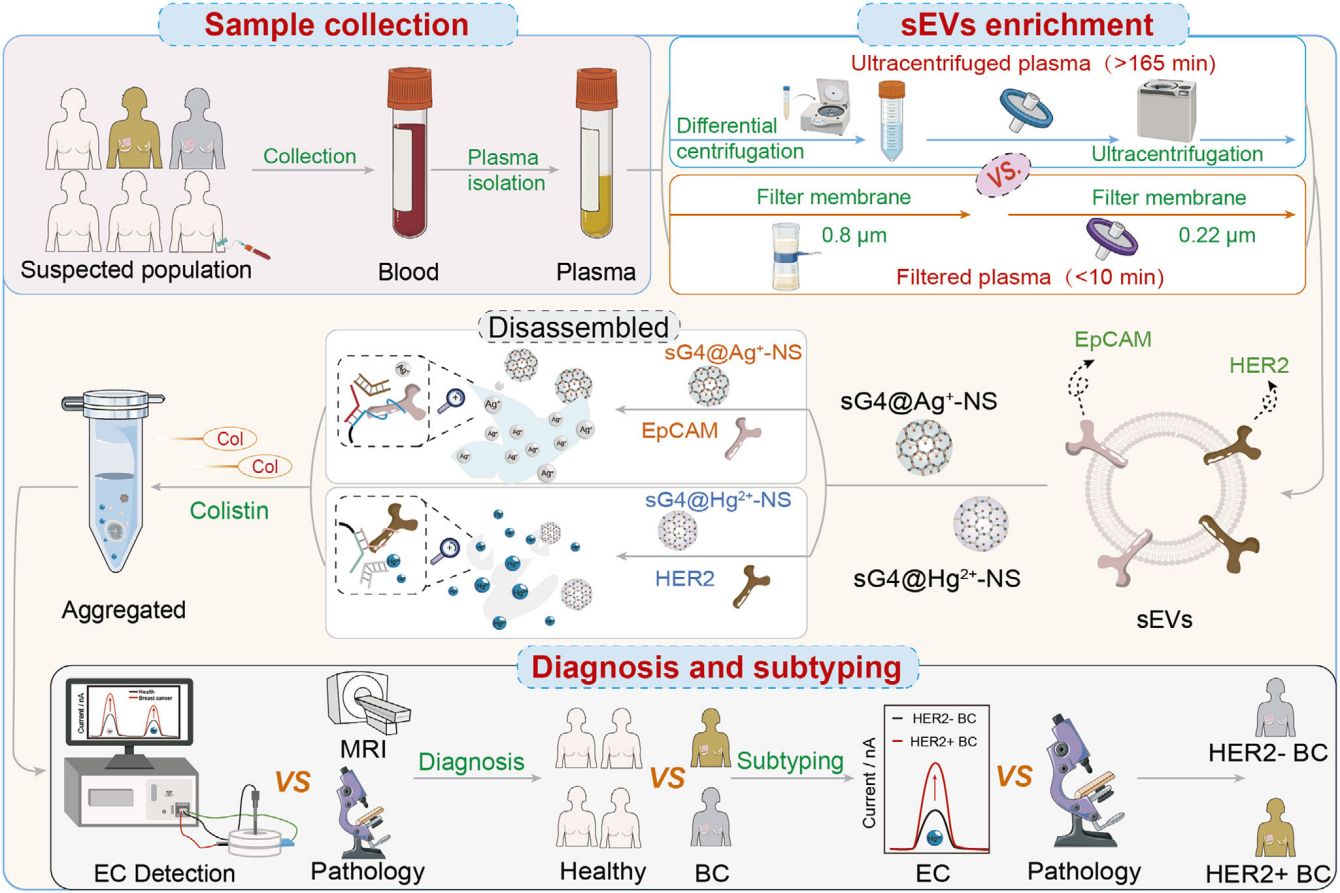
Schematic diagram of controllable cascade aggregation nanomachines for exploratory breast cancer assessment and HER2‐status discrimination.

Although this sEVs detection strategy exhibits favorable performance in clinical samples, the enrichment of sEVs still heavily relies on the cumbersome and time‐consuming ultracentrifugation method, which severely restricts its practical application and promotion. Although membrane filtration is simple and time‐saving, it tends to introduce impurities that interfere with detection. This controllable cascade‐assembled nanomachine system features excellent anti‐interference capacity and stability, making it compatible with samples prepared by membrane filtration. Accordingly, the detection performances of clinical samples processed by ultracentrifugation and membrane filtration were compared using this system, verifying the membrane filtration feasibility. Subsequently, membrane filtration was adopted for sample preparation in breast cancer detection and HER2 subtyping, providing a new strategy for simple, rapid, and reliable clinical sEVs analysis.

### 2.4 Analytical Feasibility and Performance of EpCAM and HER2

The feasibility of the proposed sensing mechanism was assessed by employing EpCAM and HER2 as target analytes (Figure 4a). For EpCAM, TEM revealed that upon incubation with the targets, the structural integrity of Ag^+^‐NS (Figure 4b) was disrupted, leading to nanosphere disassembly into amorphous debris. These findings were further confirmed by ICP‐MS analysis. As presented in Figure 4c, Ag^+^ concentration in the filtrate increased following target addition, confirming the disassembly of nanospheres. The same results were obtained for the Hg^2+^‐NS system (Figure 4d,e). Agarose gel electrophoresis was used to verify the constructed system. A new DNA band, characterized by a higher molecular weight, was present in lane 6, indicating the successful synthesis of Y_1‐3_‐NS. The addition of EpCAM protein to Y_1‐3_‐NS led to its disassembly. As presented in lanes 7 and 8, a series of low‐molecular‐weight products were generated, and an increase in concentration resulted in more low‐molecular‐weight products, demonstrating that nanosphere disassembly was target protein‐dependent (Figure 4f). Furthermore, the same verification strategy was extended to Y_4‐6_‐NS (Figure 4g). Specificity testing showed that only EpCAM and HER2 produced concentration‐dependent responses in their corresponding Ag^+^ and Hg^2+^, whereas abundant nontarget proteins and lipoprotein‐related components generated signals comparable to the blank (Figure S5). Collectively, these results confirmed that the target proteins can specifically trigger the collapse of the nanospheres, validating the feasibility of the signal‐on detection strategy.

**FIGURE 4:**
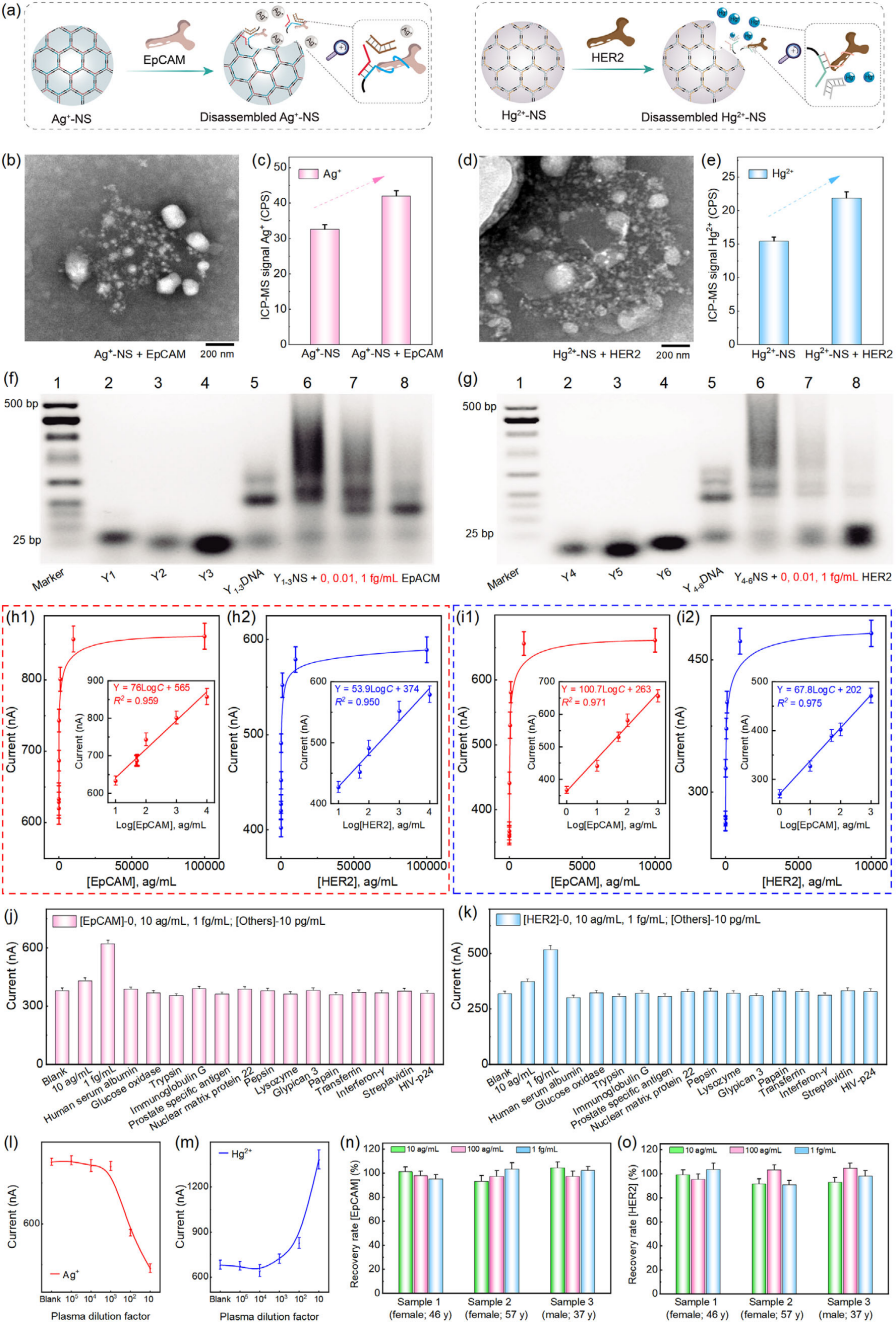
Analytical Performance of EpCAM and HER2. (a) Analysis diagram of EpCAM and HER2. (b–g) Feasibility of analysis using TEM, ICP‐MS, and agarose gel electrophoresis, respectively. (h, i) Changes in the current induced by EpCAM and HER2 without and with colistin assistance, respectively. (j, k) Selectivity analysis of the system. Plasma anti‐interference experiment of (l) Ag^+^‐NS and (m) Hg^2+^‐NS under different dilution factors of plasma. Plasma spiked recovery rates of (n) EPCAM and (o) HER2. Signal‐to‐noise ratio = (I__target_—I__blank_) / SD__blank_. Recovery (%) = (C_found_—C_original_) / C_added_ × 100%. Triple measurements calculated error bars and represented standard deviations.

To achieve optimal analytical outcomes, the experimental conditions were comprehensively optimized (Figure S6–S8). Subsequently, the analytical performance of the EC sensor for EpCAM and HER2 detection was evaluated in the presence and absence of colistin. As presented in Figure 4h1,2, the EC current showed a good linear relationship with the logarithm of target protein concentrations in the range of 10 ag/mL to 10 fg/mL (Figure S9a). With a signal‐to‐noise ratio of 3, the obtained limit of detection (LOD) was 5 ag/mL for EpCAM and 7 ag/mL for HER2, respectively [43, 44].

Next, the system with colistin assistance was studied; the EC peak was positively correlated with EpCAM and HER2 concentrations in the range from 1 ag/mL to 10 fg/mL (Figure S9b). The linear range extended to 1 ag/mL–1 fg/mL for both targets (Figure 4i1,i2). Consequently, LODs were improved to 0.5 ag/mL for EpCAM and 0.8 ag/mL for HER2. For target recognition, the colistin‐assisted strategy improved LODs by one order of magnitude compared to the non‐assisted system. Furthermore, the signal‐to‐background ratios increased from 1.4 to 1.8 for EpCAM and from 1.4 to 1.7 for HER2, respectively. These results confirmed that incorporating colistin effectively suppresses background noise, thereby boosting sensitivity and signal clarity. Time‐resolved electrochemical measurements further supported the sequential sensing mechanism. After colistin was added at 60 min, only the sG4‐functionalized nanosphere systems exhibited a marked signal decrease, whereas the unmodified and non‐G4 ssDNA‐modified controls remained stable, confirming that colistin‐induced aggregation depends on intact G4 formation (Figure S10). The robustness of the calibration models was further assessed using standard errors and 95% confidence intervals (95% CI) for the slopes and intercepts, while blank variability and the analytical noise floor were evaluated using 11 repeated no‐target measurements (Tables S8 and S9). Additional intra‐day, inter‐day, inter‐batch, and long‐term stability evaluations demonstrated good stability and acceptable reproducibility (Figure S11 and Table S10).

The sensitivity of this homogeneous EC detection platform is comparable to or even superior to that of existing methods (Table S11). Specifically, while the enzyme‐based method reported by Liu et al. achieves ag/mL‐level sensitivity, it suffers from prolonged reaction times. Conversely, the enzyme‐free strategy developed by Sweety et al., although avoiding enzymes, involves heterogeneous operations and is limited to fg/mL‐level sensitivity. Ultimately, our proposed method successfully integrates the advantages of these approaches, providing high sensitivity (ag/mL level) in an enzyme‐free, homogeneous format. This excellent performance was attributed to the synergistic effect of colistin‐mediated background reduction and signal amplification derived from the programmable self‐assembly of DNA nanospheres.

The selectivity of the method was evaluated by testing the potential interference of various proteins (Figure 4j,k). Low concentrations of the target protein (10 ag/mL and 1 fg/mL) produced a clearly elevated signal response. However, the signals of nontarget analytes at 10 pg/mL were similar to those of the blank control, which preliminarily demonstrated the favorable selectivity of the method. To establish a standardized condition for clinical plasma analysis, we evaluated the electrochemical responses of the Ag^+^‐ and Hg^2+^‐encoded nanospheres under different plasma dilution conditions. As shown in Figure 4l,m, the 10 000‐fold dilution condition stabilized the background current and matched the linear range of the assay. Previous studies have reported that plasma sEVs levels in cancer patients can reach approximately 10^9^ particles/mL [45, 46], exceeding the linear range of our assay and therefore requiring standardized dilution before detection. Therefore, this dilution condition was used consistently in subsequent clinical sample analysis, interference evaluation, and spike‐in recovery experiments. Furthermore, we spiked three different concentrations of target proteins into diluted plasma samples from three healthy volunteers (Scheme S2). The measured recoveries ranged from 91.7% to 104.7% (Figure 4n,o), confirming that this system enables accurate target detection in complex biological matrices.

### 2.5 Analytical Performance of sEVs

The feasibility of the proposed EC strategy was evaluated using sEVs derived from the cell supernatant. The workflows of the isolation protocols and analysis methods are depicted in Scheme S1a and Figure 5a. The SKBR‐3 human breast cancer cell line was selected as the model due to its high EpCAM and HER2 expression. Initially, nanoparticle tracking analysis (NTA) and TEM were used to characterize the isolated sEVs. NTA demonstrated that sEVs possessed average diameters of approximately 137 nm (Figure 5b). Furthermore, the distinctive cup‐shaped morphology of sEVs was confirmed by TEM analysis (Figure 5c). The analytical performance was subsequently assessed. EC signals gradually increased with increasing sEVs concentrations (Figure 5d). Significant linear correlations were established between the current response and the logarithm of the sEVs concentration (Figure 5e,f). Specifically, the linear range for EpCAM was 10^2–^10^7^ particles/mL (Y = 71.7Log*C* + 210, *R^2^* = 0.981), whereas for HER2, it ranged from 10^2^ to 10^6^ particles/mL (Y = 58.1Log*C* + 183, *R^2^* = 0.979). The LOD was computed as 30 particles/mL for EpCAM and 50 particles/mL for HER2 according to a signal‐to‐noise ratio of 3. sEVs derived from MCF‐7 (HER2‐low) and SKBR‐3 (HER2‐high) cells were used to assess the ability of the EC platform to discriminate between breast cancer subtypes. EC results (Figure 5g) revealed that the HER2 signal in SKBR‐3 cells was higher than that in MCF‐7 cells, whereas the intensity of EpCAM signals was similar between the two cell lines. These results were verified by Western blotting (WB). WB (Figure 5h) revealed that sEVs derived from MCF‐7 and SKBR‐3 cells expressed sEVs markers (CD63, CD9, and CD81) and high levels of EpCAM, and exhibited distinct HER2 expression profiles. HER2 was significantly overexpressed in SKBR‐3 but was negligible in MCF‐7. This differential expression pattern provides a crucial biological basis for cancer classification [47, 48]. To further evaluate phenotype‐dependent selectivity across different cell sources, sEVs derived from SKBR‐3, A549, HCT116, MCF‐10A, HaCaT, and RAW264.7 cells were analyzed. Consistent with the known high EpCAM expression in SKBR‐3, A549, and HCT116 cells, their corresponding sEVs generated markedly elevated EpCAM‐associated signals. In contrast, a pronounced HER2‐associated response was observed only for SKBR‐3‐derived sEVs, in agreement with the HER2‐high phenotype of SKBR‐3 cells. These results indicate that the platform can preliminarily differentiate cell‐source‐dependent sEVs surface‐protein (Figure 5i,j). Additional cross‐reactivity and interference tests were performed by separately mixing nontarget sEVs and interfering proteins with SKBR3‐derived sEVs. The resulting EpCAM and HER2 signals remained comparable to those obtained with SKBR3‐derived sEVs alone, indicating negligible interference from the tested components (Figure S12). Moreover, spike‐in recovery experiments using SKBR‐3‐derived sEVs in plasma samples from three healthy donors yielded recoveries of 94.2%–106.3% with RSD values below 3% for both EpCAM‐ and HER2‐associated signals, confirming acceptable quantitative accuracy and reproducibility in a complex plasma matrix (Tables S12 and S13).

**FIGURE 5:**
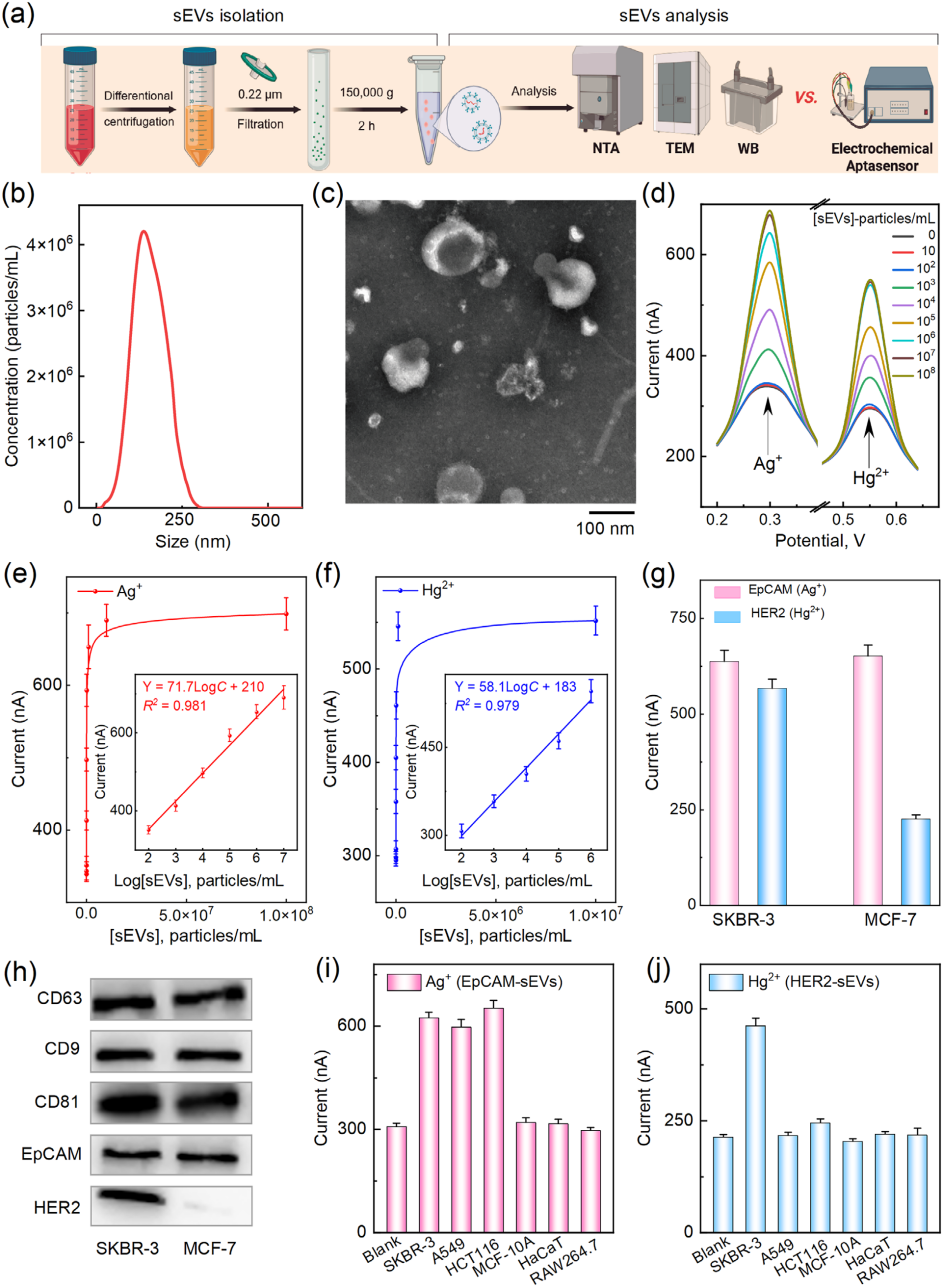
Characterization and analytical performance of the sEVs. (a) Workflow schematic for sEVs isolation and characterization. (b) NTA assessment and (c) TEM images of sEVs obtained from SKBR‐3 cells. (d, f) Changes in the EC signal at different sEVs concentrations. (g) EC results and (h) western blotting of SKBR‐3 and MCF‐7 sEVs. (i, j) Cell‐source‐dependent selectivity of the platform for EpCAM‐ and HER2‐associated sEVs signals. The sEVs concentration was set to 1 × 10^6^ particles/mL in each case. Triple measurements were calculated as error bars and represented standard deviations.

As shown in Table S14, compared with existing sEVs detection technologies, the present strategy achieves a favorable balance among sensitivity, operational simplicity, and compatibility with biological matrices. Single‐vesicle and digital platforms can provide vesicle‐level phenotypic information or multidimensional molecular readouts but typically require dedicated instruments or digital microcompartmentalization. Immunomagnetic capture and some microfluidic platforms enable efficient enrichment and specific capture but often involve solid‐phase reactions, washing steps, or antibody‐dependent. Plasmonic sensing, SERS, and surface plasmon resonance microscopy offer high sensitivity or single‐particle analysis capability but generally require functionalized substrates and dedicated optical equipment. Standardized commercial sEVs kits and routine laboratory workflows often require antibody labeling, washing steps, dedicated instruments, or longer turnaround times (Table S15). By comparison, our DNA nanomachine enables a simplified homogeneous dual‐biomarker sEVs assay within 90 min, with direct EC readout using an unmodified electrode, antibody‐free and enzyme‐free, and low reagent cost. But further standardization and clinical validation are required before clinical translation.

### 2.6 Clinical Applicability of the sEVs Analysis Method

Subsequently, the analysis platform was evaluated using sEVs isolated from plasma samples. Figure 6a compares the two methods for isolating sEVs from clinical samples. The conventional ultracentrifugation method involves cumbersome procedures, is time‐consuming (> 165 min), and relies on bulky equipment. In contrast, membrane filtration enables efficient isolation in 10 min through a simpler operation. According to previous reports, this study used a dual‐pore‐size sequential filtration system [49, 50, 51]. Impurities and cell debris were first removed using a 0.8 µm filter, and sEVs were further enriched using a 0.22 µm filter to simplify the pretreatment workflow. The sEVs isolated by both methods were characterized by size distribution analysis and morphological observation.

**FIGURE 6:**
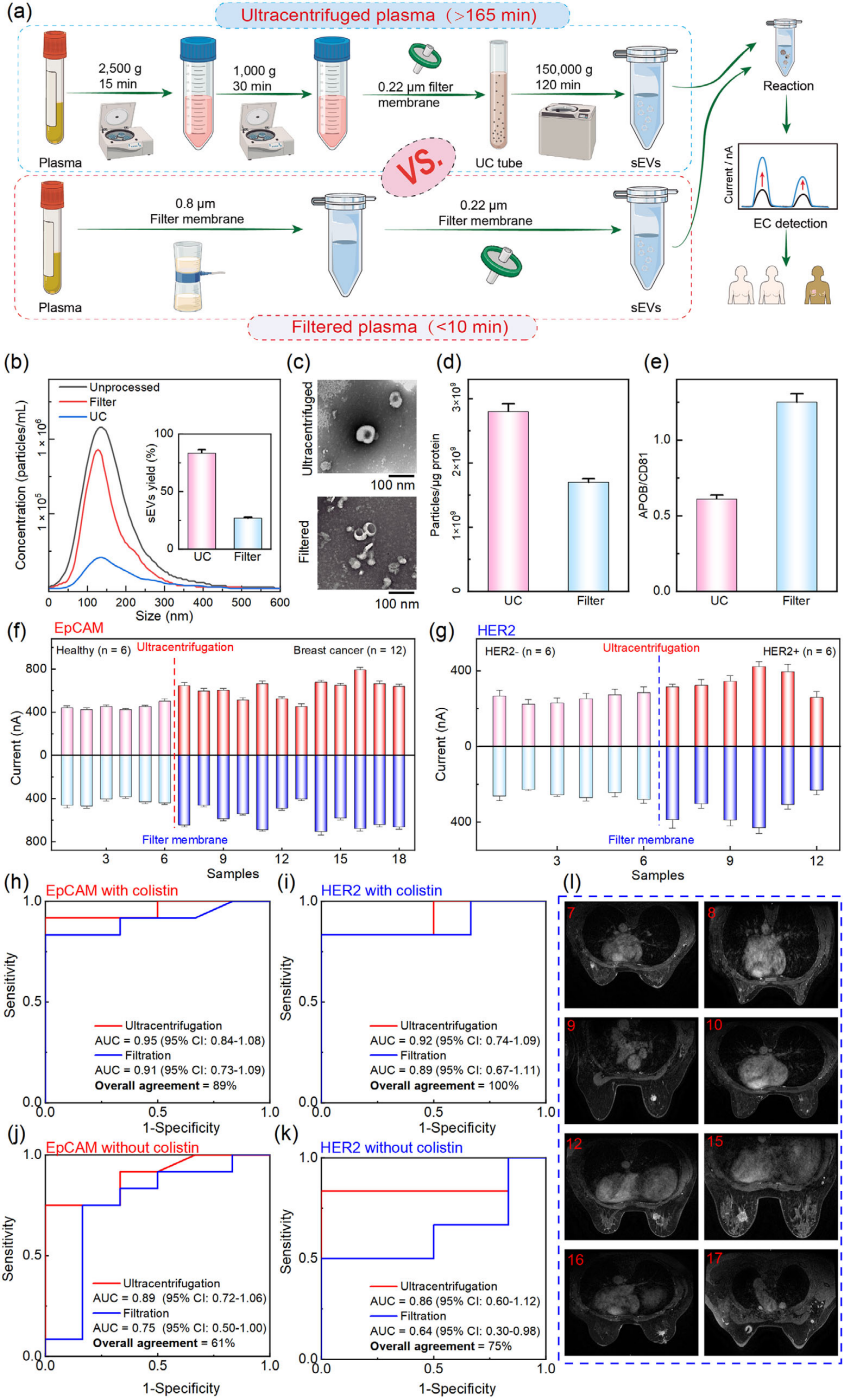
Comparison between the simplified filtration strategy and ultracentrifugation for sEVs enrichment. (a) Schematic workflow comparing ultracentrifugation and rapid filtration methods in this study for sEVs enrichment and subsequent sensing detection. (b) Size distribution profiles (left) and particle yield of two isolation methods (right). (c) TEM results of sEVs isolated by ultracentrifugation and filtration. (d) Particles/protein ratio of two isolation methods (e) Purity assessment based on the APOB/CD81 ratio determined by Western blot densitometry. Bar chat of (f) EpCAM and (g) HER2 signals after ultracentrifugation or membrane filtration. ROC analyses comparing the two pretreatment methods with colistin (h‐i) and without colistin (j‐k). (l) Clinical MRI results. Triple measurements calculated error bars and represented standard deviations.

We first systematically compared the key parameters of sEVs isolated by the two methods. NTA quantification revealed that the filtration method yielded significantly more particles than ultracentrifugation (Figure 6b), and TEM imaging confirmed that both methods effectively isolated sEVs with typical cup‐shaped morphology (Figure 6c). Regarding the particle/protein ratio, ultracentrifugation was approximately 1.6‐fold higher than filtration (Figure 6d). However, since NTA cannot discriminate sEVs from non‐vesicular particles, we further performed WB analysis for the sEVs marker CD81 to evaluate purity. Under equal total protein loading, the CD81 signal of ultracentrifugation‐isolated sEVs was 43% higher than that of the filtration method, indicating significantly superior relative purity of sEVs per unit protein. Meanwhile, WB analysis of apolipoprotein B (APOB) as a typical contamination marker showed that the filtration method exhibited higher lipoprotein contamination than ultracentrifugation (Figure 6e). Despite its lower purity relative to ultracentrifugation, the filtration method was employed in our study due to its ease of use and compatibility with our highly robust detection platform, which tolerates moderate impurity levels.

The feasibility of membrane filtration for sEVs enrichment was further evaluated by analyzing plasma samples from 6 healthy volunteers and 12 patients with breast cancer. sEVs enrichment in parallel by filtration and ultracentrifugation was quantified using a controllable aggregation metal‐nanosphere detection platform (Table S16). As shown in Figure 6f, EpCAM‐associated EC signals were higher in patients with breast cancer than in healthy controls. In contrast, Figure 6g showed that HER2‐associated EC signals were higher in HER2‐positive than in HER2‐negative patients. These trends were consistently observed for both ultracentrifugation‐ and membrane filtration‐processed samples. In this method‐comparison cohort, the combination of membrane filtration with our sensitive, anti‐interference sensing platform showed preliminary analytical performance comparable to ultracentrifugation. Based on the AUC point estimates and a prespecified non‐inferiority margin of ΔAUC < 0.10, the filtration‐based workflow met the predefined criterion for both EpCAM‐based breast cancer discrimination (ΔAUC = 0.04) and HER2‐status discrimination (ΔAUC = 0.03), with classification agreement rates of 89% and 100%, respectively (Figure 6h,i) [52, 53]. In the absence of colistin, both agreement and AUC comparability declined, and the non‐inferiority criterion was no longer met (Figure 6j,k). These results preliminarily support that rapid membrane filtration, when coupled with the present high‐sensitivity detection platform, can satisfy the analytical requirements of targeted sEVs‐associated signal assessment without performing worse than ultracentrifugation in this cohort. Moreover, the experimental results from these samples demonstrated high concordance with the clinical MRI diagnosis (Figure 6l).

After validating the feasibility of the filtration‐based isolation strategy, we comprehensively evaluated the clinical discrimination performance of the proposed sensing platform. Plasma samples from a cohort of 63 patients with breast cancer and 22 nonmalignant controls, including 8 patients with benign breast disease and 14 healthy individuals, were processed using the streamlined filtration workflow (Figure 7a and Table S17). EC signals for EpCAM‐sEVs were elevated in the breast cancer cohort compared with both healthy controls and patients with benign breast diseases (Figure 7b,c), with statistically significant differences observed between groups. A consistent trend was observed for HER2 detection (Figure 7d,e). To further assess the diagnostic performance of the system, we conducted a receiver operating characteristic (ROC) curve analysis. The optimal cutoff values were determined using the Youden index, and diagnostic metrics were calculated accordingly [54]. For EpCAM detection (Figure 7f), the system achieved a diagnostic sensitivity of 81.0% (51/63, 95% CI: 69.1%–89.8%), a specificity of 100.0% (22/22, 95% CI: 84.6%–100.0%), and an area under the curve (AUC) of 0.94 (95% CI: 0.91–0.99). ROC analysis (Figure 7g) showed that HER2 detection achieved a sensitivity of 82.5% (52/63, 95% CI: 70.9%–90.9%), a specificity of 72.7% (16/22, 95% CI: 49.8%–89.3%), and an AUC of 0.82 (95% CI: 0.73–0.92).

**FIGURE 7:**
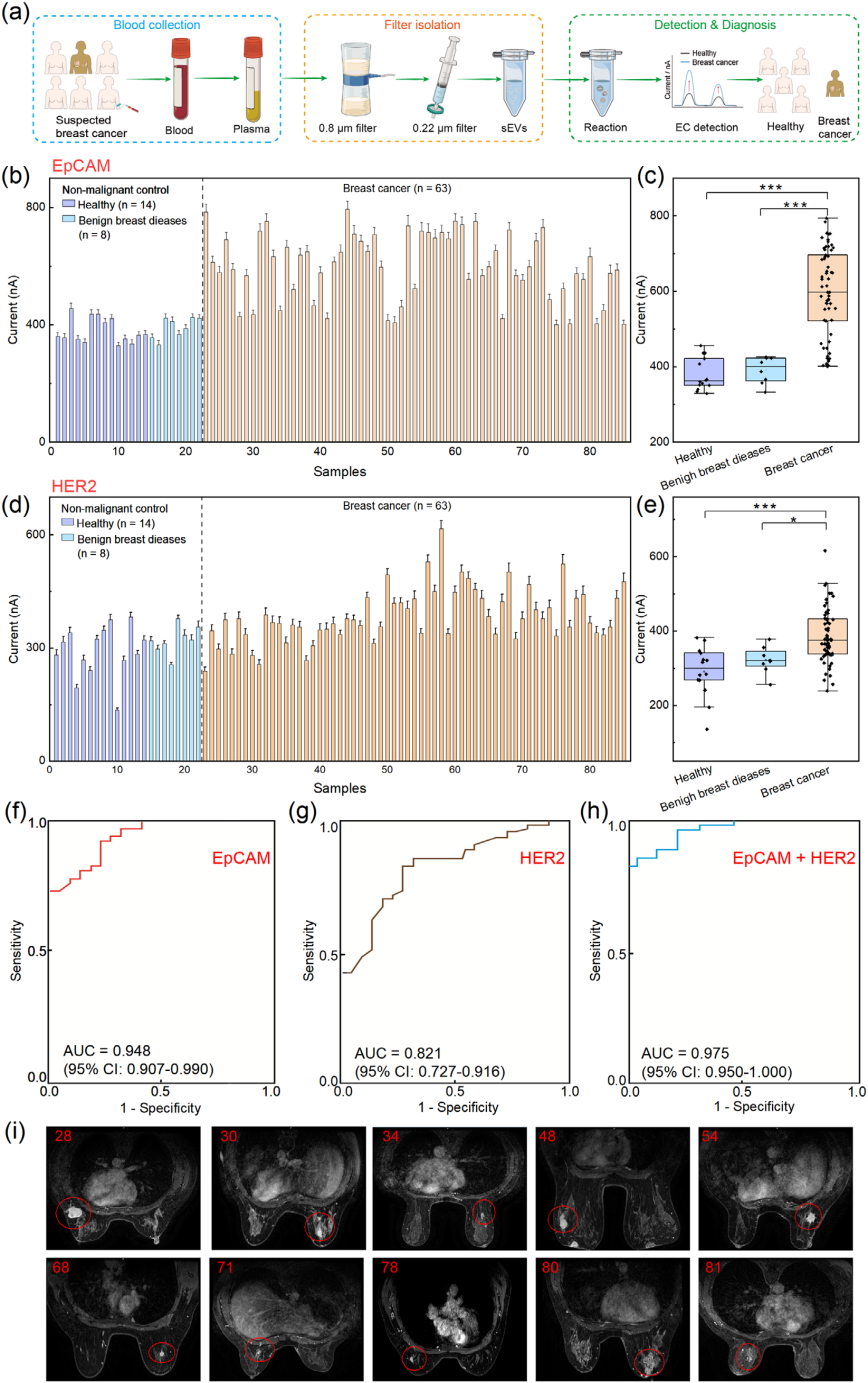
Preliminary evaluation of the discrimination performance of this detection platform for breast cancer. (a) Workflow for sEVs enrichment and EC detection of clinical samples. (b, c) EpCAM EC signal distributions via bar chart and box plot, respectively. (d, e) HER2 EC signal distributions in the same cohorts. (f–h) ROC analysis of EpCAM, HER2, and the combination of EpCAM and HER2. (i) Clinical MRI results. Statistical significance was evaluated using the Mann–Whitney U test. Data are presented as mean ± SD. Statistical significance: ^*^ *p* < 0.05, ^**^ *p* < 0.01, and ^***^ *p* < 0.001. 95% CI: 95% confidence interval. Triple measurements calculated error bars and represented standard deviations.

Although approximately 60% of patients with breast cancer in this study were HER2‐negative (HER2‐), HER2 still exhibited certain diagnostic efficacy in distinguishing patients with breast cancer from healthy individuals. The main reason was that HER2‐negative breast cancer mostly presents a low HER2 expression status, whose level remains significantly higher than that in healthy people, which was consistent with previous studies [55]. Notably, the combined EpCAM‐HER2 model (Figure 7h) achieved an AUC of 0.975 (95% CI: 0.950–1.000), with a sensitivity of 88.9% (56/63, 95% CI: 78.4%–95.4%) and a specificity of 100.0% (22/22, 95% CI: 84.6%–100.0%). Additionally, the results of this method were significantly consistent with MRI diagnosis results (Figure 7i).

The above preliminary findings verify that the combination of facile membrane‐filtration pretreatment and our self‐developed controllable aggregation metal‐DNA nanomachine enables proof‐of‐concept detection of breast cancer‐related sEVs. Compared with high‐purity separation approaches such as meso‐macroporous hydrogel capture and immunomagnetic affinity isolation, the sequential membrane filtration adopted in this work has inherent limitations in sEVs purity and the removal efficiency of non‐vesicular contaminants (Table S18) [56, 57, 58]. Nevertheless, this method features simplified operation, fast sample enrichment, and no requirement for customized separation materials or dedicated separation instruments. In future work, integrating our detection platform with advanced purification techniques including hydrogel capture and immunomagnetic isolation will likely further reduce interference from non‐vesicular components, as well as improve quantitative accuracy, analytical specificity, and the confidence level for attributing biomarkers specifically to sEVs.

Critically, as a proof‐of‐concept system, this assay showed preliminary ability to distinguish HER2 status. Figure 8a illustrates the streamlined workflow, including rapid sEVs enrichment by filtration followed by exploratory HER2‐status assessment. Among 63 patients with breast cancer, 21 were confirmed to be HER2‐positive (HER2+), and 42 were HER2‐, using immunohistochemistry pathological results as the gold standard. As depicted in the bar chart (Figure 8b), despite several outliers (marked by green arrows), the EC detection platform yielded significantly elevated signal intensities for the HER2+ cohort compared to the HER2‐ cohort. This distinct discrepancy in expression profiles was further visualized using a heatmap (Figure 8c), where darker colors demarcate higher EC signals for HER2 detection. Box plot analysis confirmed that the difference in EC signals between the two molecular subtypes was highly significant (Figure 8d; *p* < 0.001). The subtype determination performance was further evaluated using ROC curve analyses and a confusion matrix (Figure 8e,f). In this cohort, the platform achieved an AUC of 0.902 (95% CI: 0.804–1.000) for exploratory HER2‐status discrimination, with a sensitivity of 90.5% (19/21, 95% CI: 69.6%–98.8%) and a specificity of 88.1% (37/42, 95% CI: 74.4%–96.0%). Post hoc power analysis and repeated stratified five‐fold cross‐validation supported the internal robustness of the combined diagnostic models, while independent external validation remains necessary (Table S19). In summary, the controllable aggregation metal‐DNA nanomachine detection platform constructed in this work, combined with a simple filter‐based plasma pretreatment protocol, provides a preliminary strategy for tumor liquid biopsy research and exploratory HER2‐status assessment, while larger independent cohorts are required before clinical decision‐making applications can be considered.

**FIGURE 8:**
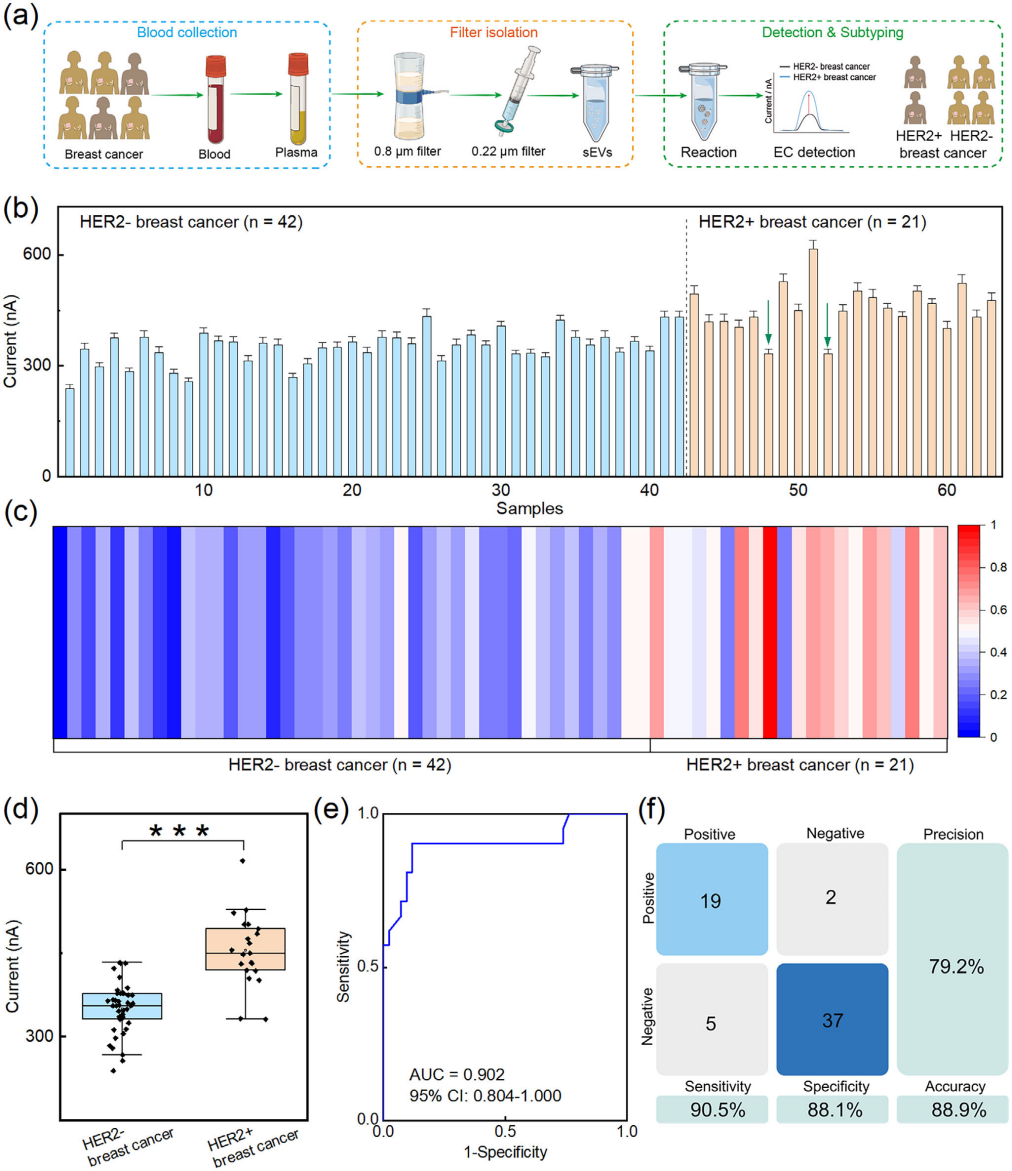
Exploratory evaluation of HER2‐status discrimination in breast cancer samples. (a) Workflow for sEVs isolation and EC detection of clinical samples. (b–d) HER2 EC signal distributions via bar chart, heatmap, and box plot, respectively. (e, f) ROC curve and confusion matrix for HER2 subtyping. Statistical significance was evaluated using the Mann–Whitney U test. Data are presented as mean ± SD. Statistical significance: ^*^ *p* < 0.05, ^**^ *p* < 0.01, and ^***^ *p* < 0.001. 95% CI: 95% confidence interval. Triple measurements calculated error bars and represented standard deviations.

## 3 Conclusion

In summary, we constructed a simple, controllable aggregation‐ion‐programmed nanomachine detection platform. By combining a dual‐membrane filtration strategy with electrochemical detection, this platform enabled exploratory analysis of EpCAM/HER2‐positive sEVs‐associated signals in breast cancer samples. Distinct from conventional sEVs analysis strategies, our colistin‐mediated “active background silencing” mechanism effectively suppresses nonspecific signal leakage while preserving high target responsiveness. Upon specific sEVs‐aptamer recognition, DNA nanostructures undergo structural disassembly, triggering the release of Ag^+^ and Hg^2+^ for simultaneous and sensitive EC quantification of EpCAM and HER2. In the proof‐of‐concept cohort, the platform showed preliminary concordance with imaging and pathological assessments.

The superior effects were achieved by the synergistic combination of G4‐functionalized nanospheres with colistin‐mediated supramolecular assembly to construct a structurally stable and noise‐controllable nanomachine. Compared with previous enzyme‐free strategies, this design improves probe stability and suppresses nonspecific ion leakage under the optimized diluted‐plasma detection workflow, thereby enhancing analytical performance in terms of “signal on” and “noise suppression.” The resulting homogeneous assay integrates isolation, recognition, amplification, and noise regulation into a single platform, eliminating the need for ultracentrifugation and reducing the operational complexity. Moreover, dual‐target detection was achieved via orthogonal metal‐ion encoding without signal crosstalk, ensuring high analytical fidelity. This system features a highly modular design, enabling multiplexed detection of multiple biomarkers by introducing DNA nanospheres with distinct functionalities. Meanwhile, the detection targets and corresponding disease types can be flexibly switched simply by replacing the aptamers loaded inside the nanospheres. Notably, the as‐prepared nanomaterials exhibit storage stability and long‐term detection reproducibility, which may provide a basis for further development of stable and scalable assay strategies.

Despite its promising performance, several limitations and future directions must be considered. Firstly, larger‐scale and multi‐center studies are required to further validate clinical utility and standardized implementation. The HER2‐status analysis should also be interpreted cautiously because HER2‐positive cases were limited, precluding reliable subgroup analysis. Although all patients were non‐treatment before blood collection, tissue‐sEVs HER2 discordance may still occur due to tumor heterogeneity and temporal changes. Future studies should include larger HER2‐positive cohorts and longitudinal follow‐up. Secondly, expanding the application of this platform to a broader range of cancer types and biomarkers is essential to establish its generalizability and versatility. Thirdly, this assay relies on electrochemical workstations for signal readout. In follow‐up work, compact handheld electrochemical detectors will be developed to match point‐of‐care requirements and facilitate on‐site rapid clinical screening. Furthermore, Hg^2+^ nanospheres were used as a model for mechanistic investigation; their heavy metal toxicity restricts clinical translation, and low‐toxicity, biocompatible metal nanomaterials such as Cu^2+^ and Fe^2+^ nanospheres can be adopted as substitutes in subsequent studies. In addition, although the filtration method enables high sEVs recovery efficiency, abundant interfering contaminants are co‐isolated simultaneously. Even though the sensing system showed acceptable anti‐interference performance under the optimized diluted‐plasma workflow and preliminary diagnostic discrimination, the composite filter strategy requires further optimization. As an ensemble assay, the present platform cannot independently distinguish changes in marker‐positive sEVs abundance from changes in antigen density per vesicle, which will require complementary particle counting and single‐vesicle phenotyping in future studies.

## 4 Methods

### 4.1 Ion‐Programmed Nanomachine Synthesis

(1) sG4@Ag^+^‐NS: 2 µL of 40 µM Y1, Y2, Y3, and sG4 (sG4a and sG4a') were mixed with 240 µL of 3‐(*N*‐morpholino) propanesulfonic acid (MOPS) buffer (10 mM, 100 mM NaNO_3_, pH 7.4), followed by the sequential addition of 30 µL of 600 µM AgNO_3_ and 20 µL of 80 mM KNO_3_ solution. After vortexing for 10 s, the mixture was heated at 95°C for 5 min and then cooled to room temperature (RT) for 3 h, enabling self‐assembly into sG4@Ag^+^‐NS.

(2) sG4@Hg^2+^‐NS: 2 µL of 40 µM Y4, Y5, Y6, and sG4 (sG4e and sG4e') were mixed with 240 µL of MOPS buffer, followed by the sequential addition of 30 µL of 600 µM mercury (Hg^2+^) standard solution and 20 µL of 60 mM KNO_3_ solution. Following 10 s of vortexing, the mixture was heated at 95°C for 5 min and incubated at RT for 3 h to facilitate self‐assembly into sG4@Hg^2+^‐NS.

### 4.2 Steps for EpCAM and HER2 Analysis

Approximately 50 µL EpCAM and 50 µL HER2 at different concentrations were added to 265 µL of 10 mM MOPS buffer, followed by adding 20 µL sG4@Ag^+^‐NS and 15 µL sG4@Hg^2+^‐NS. The mixture was then incubated at RT for 60 min. Subsequently, 20 µL of 20 µM colistin was added to the reaction solution, which was conducted at room temperature for 30 min.

### 4.3 Cell Lines and Antibodies

SKBR‐3 (RRID: CVCL_0033), MCF‐10A (RRID: CVCL_0598), HaCaT (RRID: CVCL_0038), HCT116 (RRID: CVCL_0291), and A549 (RRID: CVCL_0023) cell lines were obtained from Xiamen Immocell Biotechnology Co., Ltd. MCF‐7 cells (RRID: CVCL_0031) were purchased from Zhanjiang Tongbo Medical Technology Co., Ltd. RAW 264.7 cells (RRID: CVCL_0493) were acquired from the American Type Culture Collection (ATCC, Manassas, VA, USA). Anti‐Apolipoprotein B (APOB, RRID: AB_10732938), anti‐CD63 (RRID: AB_2783831), anti‐CD9 (RRID: AB_2878706), anti‐CD81 (RRID: AB_2880995), and anti‐EpCAM (RRID: AB_10693684) were purchased from Proteintech (Rosemont, IL, USA). The anti‐HER2 antibody (RRID: AB_2532480) was ordered from Thermo Fisher Scientific (Waltham, MA, USA). Other reagents were available in the Supporting Information.

### 4.4 Analysis Steps of sEVs

Approximately 200 µL of sG4@Ag^+^‐NS, 150 µL of sG4@Hg^2+^‐NS, and 1 mL of sEVs at various concentrations were mixed with 2.65 mL of 10 mM MOPS solution. The resulting mixtures were incubated at RT for 60 min, followed by the addition of 200 µL of 20 µM colistin and a subsequent reaction for 30 min. Finally, differential pulse voltammetry (DPV) tests were performed on a CHI 660E electrochemical workstation equipped with a standard three‐electrode system, where Ag/AgCl served as the reference electrode, Pt as the counter electrode, and bare ITO as the working electrode. Notably, this homogeneous electrochemical sensing system requires no electrode surface modification. Signals were recorded under DPV mode with potential scanning ranging from −0.1 to 0.7 V. The same peak‐picking, baseline‐correction, and blank‐subtraction settings were consistently applied to calibration standards, blank controls, spiked samples, and clinical samples.

### 4.5 Pretreatment Steps for Clinical Samples

This study, which enrolled 14 healthy controls, 8 benign breast disease patients, and 63 breast cancer patients from West China Hospital, Sichuan University (Chengdu, China; Ethical Approval No. 20221489), was executed in adherence to the Declaration of Helsinki, and written informed was obtained from all participants prior to sample collection, indicating their informed agreement to participate. For the breast cancer group, the enrollment criteria were strictly restricted to newly diagnosed patients without prior treatment. All patients were definitively diagnosed via core needle biopsy pathology, and no surgical intervention, neoadjuvant therapy, or other relevant clinical management had been administered before enrollment. Patients with benign breast diseases were those with biopsy‐confirmed nonmalignant breast nodules. Healthy controls consisted of individuals with no obvious abnormalities observed in imaging and laboratory examinations. All participants were recruited from the same medical center, all testing was conducted under single‐blind conditions, and all samples were collected by a dedicated researcher to control biases. Indeterminate cases with unclear diagnostic findings were excluded in accordance with standardized criteria.

Venous blood (5 mL) was drawn from all participants using a single‐use closed EDTA‐anticoagulated vacuum blood collection kit. The procedure for sEVs isolation via ultracentrifugation was as follows: Two rounds of centrifugation at 2500 g for 15 min were performed on whole blood samples to isolate platelet‐poor plasma (Scheme S1b). Subsequent centrifugation of the collected plasma under the same parameters was conducted to obtain purified platelet‐poor plasma, which was stored at −80°C until further use. The procedure for sEVs isolation via filter membrane was as follows: Plasma was separated by centrifuging whole blood samples at 2500 g for 15 min. Subsequently, a dual‐pore‐size sequential filtration system was used to separate the plasma. Impurities and cell debris were first removed using a 0.8 µm filter, followed by the precise isolation of sEVs using a 0.22 µm filter. The isolated sEVs were used for subsequent analyses.

## Author Contributions

**Zhihao Zhang**: data curation, Writing – original draft. **Binxu Qiu**: writing – original draft. **Jie Chen**: supervision, writing – review and editing, funding acquisition. **Piaopiao Chen**: supervision, writing – review and editing, funding acquisition. **Zijing Liu**: data curation, writing – original draft. **Xiangyue Meng**: resources, writing – original draft. **Yu Jiang**: writing – review and editing. **Pengjun Jiang**: data curation, writing – original draft. **Jie Qu**: software, writing – original draft.

## Conflicts of Interest

The authors declare no conflicts of interest.

## Supporting information




**Supporting File**: advs77177‐sup‐0001‐SuppMat.doc.
