# Supplementary material for: Controllable Cascade Aggregation Ion Programmed Nanomachines and Simple Isolation Enable Tumor‐Derived Small Extracellular Vesicles Detection for Enhanced Breast Cancer Assessment
Source: Adv Sci (Weinh). 2026 Aug 14;13(61):e77177. doi: 10.1002/advs.77177 (PMC13474031; doi:10.1002/advs.77177)
Supplement: Supplementary file 1 [file ADVS-13-e77177-s001.doc]

**Supporting Information**

**Controllable Cascade Aggregation Ion Programmed Nanomachines and Simple Isolation Enable Tumor-Derived Small Extracellular Vesicles Detection for Enhanced Breast Cancer Assessment**

*Zhihao Zhang^#^, Zijing Liu^#^, Xiangyue Meng, Pengjun Jiang, Jie Qu, Yu Jiang, Binxu Qiu, Jie Chen*, and Piaopiao Chen**

Z. H. Zhang, Z. J. Liu, X. Y. Meng, P. J. Jiang, J. Qu, Prof. Y. Jiang, B. X. Qiu, Prof. J. Chen and P. P. Chen

Department of Laboratory Medicine, Med+X Center for Manufacturing, Department of General Surgery, State Key Laboratory of Biotherapy, Department of Medical Oncology, State Key Laboratory of Respiratory Health and Multimorbidity, West China Hospital, Sichuan University, Chengdu, Sichuan, 610041, China

Breast disease center, West China Hospital, Sichuan University, Chengdu, Sichuan, 610041, China

E-mails: *chenjiedoctor@wchscu.cn; chenpp0828@wchscu.cn.*

^#^Zhihao Zhang and Zijing Liu contributed equally to this work.

Content

Title	S-1

Content	S-2

Materials and reagents	S-4

Table S1. Sequences of oligonucleotides of Ag^+^-nanospheres and Hg^2+^-nanospheres used in the study	S-5

Table S2. Sequences of split G-quadruplex used in the sG4@Ag^+^-nanospheres ..........................S-6

Table S3. Sequences of split G-quadruplex used in the sG4@Hg^2+^-nanospheres	S-6

Agarose Gel Electrophoresis of nanospheres	S-6

Agarose Gel Electrophoresis of EpCAM Analysis	S-7

Agarose Gel Electrophoresis of HER2 Analysis	S-8

Table S4. Sequences of oligonucleotides used for agarose gel electrophoresis of EpCAM	S-9

Table S5. Sequences of oligonucleotides used for agarose gel electrophoresis of HER2	S-9

Table S6. DNA sequences used in specificity experiments.	S-9

Figure S1. The chemical structure of [colistin](https://www.chemsrc.com/en/cas/108750-13-6_955452.html)...............................................................................S-10

Instruments...................................................................................................................................S-10

Isothermal titration calorimetry....................................................................................................S-11

Figure S2. ITC analysis of the interaction between non-G4 single-stranded DNA (ssDNA) and colistin..........................................................................................................................................S-12

Figure S3. UV-vis absorption spectra of G4 under different incubation systems........................S-12

Figure S4. Circular dichroism spectra..........................................................................................S-13

Cell Recovery	S-13

Cell Culture, passage and sEVs isolation	S-14

Scheme S1. Workflow schematic for the isolation and enrichment of sEVs	S-16

sEVs Isolation of Clinical Plasma Samples.	S-16

Nanoparticle tracking analysis	S-16

Western blotting	S-17

Table S7. Operational Parameters of ICP-MS	S-18

Figure S5. Specific binding between target protein and nanospheres	S-18

Optimization of EpCAM and HER2 Analysis Conditions	S-19

Figure S6. Optimization of conditions based on self-assembly of sG4@Ag^+^-NS..	S-20

Figure S7. Optimization of conditions based on self-assembly of sG4@Hg^2+^-NS......................S-21

Figure S8. Optimization of EpCAM and HER2 analysis conditions	S-22

Figure S9. DPV responses without or with colistin	S-23

Figure S10. Time-dependent kinetic electrochemical curve	S-23

Figure S11. Stability assay	S-24

Figure S12. Evaluation of cross-reactivity and interference	S-24

Table S8. Calibration uncertainty of the calibration curve.	S-24

Table S9. Noise-floor analysis of the electrochemical sensing system..	S-25

Table S10. Relative Standard Deviation (RSD) values of long-term reproducibility...	S-25

Serum stability and spiked recovery experiments	S-25

The calculation of limit of detection ............................................................................................S-26

Table S11. Comparison of methods for the determination of EpCAM and HER2 .....................S-27

Table S12. Determination of EpCAM-sEVs in spiked plasma samples (n=3).............................S-29

Table S13. Determination of HER2-sEVs in spiked plasma samples (n=3)................................S-29

Table S14. Comparison of various methods for detecting sEVs...................................................S29

Table S15. Comparison of commercial and routine workflows for EVs-related analysis.............S31

Table S16. Information of clinical samples used for comparison between ultracentrifugation and filtration methods...........................................................................................................................S33

Table S17. Clinical diagnosis and electrochemical test results of clinical cancer patients............S33

Table S18. Comparison of emerging methods for isolation of sEVs............................................S35

Table S19. Internal cross-validation performance for breast cancer diagnosis and HER2-status discrimination................................................................................................................................S36

Statistical analysis..........................................................................................................................S37

The Calculation of Sensitivity and Specificity...............................................................................S37

References......................................................................................................................................S38

**Materials and reagents.**

All oligonucleotides with different sequences were synthesized and purified by Sangon Biotechnology Co.; Ltd (Shanghai, China). The sequences of the oligonucleotides used in this work were shown in Tables S1-S3. AgNO_3_ and mercury (Hg^2+^) standard solution were ordered from Aladdin Reagent Co. (Shanghai, China). Sodium nitrate (NaNO_3_), HCl, KNO_3_ and NaOH were purchased from Kelong Chemical Reagents (Chengdu, China). Epithelial Cell Adhesion Molecule (EpCAM) was purchased from Novoprotein Scientific Inc. Human epidermal growth factor receptor 2 (HER2) was ordered from Sangon Biotechnology Co.; Ltd (Shanghai, China). Transferrin, papain, trypsin, streptavidin (SA), human serum albumin (HSA), human immunodeficiency virus (HIV) p24, prostate specific antigen (PSA), glucose oxidase (GOD), interferon-*γ*, thrombin, immunoglobulin G (IgG), nuclear matrix protein 22 (NMP-22), glypican 3 (GPC3), low-density lipoprotein (LDL), high-density lipoprotein (HDL), Apolipoprotein B, and lysozyme were ordered from Sigma-Aldrich (St. Louis, MO, USA). Colistin sulfate was purchased from MedChemExpress (MCE, NJ, USA). 3-(*N*-Morpholino) propanesulfonic acid (MOPS) was purchased from Solarbio Technology Co.; Ltd (Beijing, China). All working solutions were prepared with a MOPS buffer solution (10 mM, 100 mM NaNO_3_, pH 7.4). For membrane-related products, 0.22 μm nylon 6 syringe filters were purchased from Tianjin Jinteng Experimental Equipment Co., Ltd. (China). The 0.8 μm filter membrane was purchased from Delv New Material Technology Co., Ltd (Haining, Zhejiang, China). McCoy's 5A Medium was purchased from Thermo Fisher Technology Co.; Ltd (China). DMEM high glucose medium was purchased from Cytiva (Logan, UT, USA). Ultra-clear centrifuge tubes (25 × 89 mm, 38.5 mL, sterile, open-top thin wall) was purchased from Beckman Coulter (Indianapolis, IN, USA). The 0.45 μM PVDF transfer membrane was purchased from Merck Millipore Ltd. Phosphate buffered saline (PBS) was ordered from Corning (New York, USA). Fetal Bovine Serum (FBS) was ordered from ExCell Bio. (Shanghai, China). All reagents used in this work were of analytical or higher grade and used without further purification. Water used in all experiments was purified by a water purification system (Chengdu Ultrapure Technology Co.; Ltd.; Chengdu, China) with a resistivity of 18.25 MΩ·cm. Whole blood of lung cancer patient and healthy volunteer samples were donated from the West China Hospital of Sichuan University (Chengdu, China, approval number: 20221489). All solutions were stored at 4 °C in a refrigerator until use.

**Table S1.** Sequences of oligonucleotides of Ag^+^-nanospheres and Hg^2+^-nanospheres used in the study

| Names | Sequences (5'-3') |
| --- | --- |
| Y1 | CCCCCCCCTGAAGGTTCGTTGTTTCGGTGGGTGTAGACTCTTTAGAAGAGATACAGATTTTGGGAATG |
| Y2 | CCCCCCAATACGTCAATGAGCGAAACAACGAAGGTTATATATTCC |
| Y3 | CCCCCCTAA AGAGTCTACTTGACGTATTCCTATTATTGTCC |
| Y4 | TTTTTTATGCAGCGGTGTGGGGATT |
| Y5 | TTTTAATCCCCTATGTGATTCGGTACTACAGTA |
| Y6 | TTTTGAATCACCTGCATGGTCAGACTCTT |

**Table S2.** Sequences of oligonucleotides used for agarose gel electrophoresis of Ag^+^-nanospheres.

| Name | Sequence (5’-3’) |
| --- | --- |
| Y1 | TAGATCTATGAAGGTTCGTTGTTTCGGTGGGTGTAGACTCTTTAGAA GAGATACAGATTTTGGGAATG |
| Y2 | GCATGCAATACGTCAATGAGCGAAACAACGAAGGTTATATATTCC |
| Y3 | GCATGCTAA AGAGTCTACTTGACGTATTCCTATTATTGTCC |

**Table S3.** Sequences of oligonucleotides used for agarose gel electrophoresis of Hg^2+^-nanospheres

| Name | Sequence (5’-3’) |
| --- | --- |
| Y4 | ATTAATATGCAGCGGTGTGGGGATT |
| Y5 | AATTAATCCCCTATGTGATTCGGTACTACAGTA |
| Y6 | AATTGAATCACCTGCATGGTCAGACTCTT |

**Agarose Gel Electrophoresis**

Weigh 2.5 g agarose in advance into 100 mL of 1 × TAE solution, mix well, and heat in a microwave oven until the solution is uniform and transparent. Then add 10 μL 4SGeIRed nucleic acid dye to the above mixture, mix well and pour it into the membrane to form 2.5% agarose gel. In electrophoretic assay, each sample channel ended up containing 15 μL sample and 5 μL 6 x Loading buffer. Electrophoresis was performed in a 1 x TAE buffer and energized at 100 V for 70 min. Each lane of the gel represented different samples as detailed in subsequent descriptions.

Figure 1c:

Lane 1: Marker (25-500 bp)

Lane 2: Y1 (5 μΜ)

Lane 3: Y2 (5 μΜ)

Lane 4: Y3 (5 μΜ)

Lane 5: Y_1-3_DNA (5 μΜ)

Lane 6: Y_1-3_-NS (5 μΜ)

Figure 1d:

Lane 1: Marker (25-500 bp)

Lane 2: Y4 (5 μΜ)

Lane 3: Y5 (5 μΜ)

Lane 4: Y6 (5 μΜ)

Lane 5: Y_4-6_DNA (5 μΜ)

Lane 6: Y_4-6_-NS (5 μΜ)

**Agarose Gel Electrophoresis of EpCAM Analysis.**

Weigh 2.5 g agarose in advance into 100 mL of 1 × TAE solution, mix well, and heat in a microwave oven until the solution is uniform and transparent. Then add 10 μL 4SGeIRed nucleic acid dye to the above mixture, mix well and pour it into the membrane to form 2.5% agarose gel. In electrophoretic assay, each sample channel ended up containing 15 μL sample and 5 μL 6 x Loading buffer. Electrophoresis was performed in a 1 x TAE buffer and energized at 100 V for 70 min. Each lane of the gel represented different samples as detailed in subsequent descriptions.

Figure 4f:

Lane 1: Marker (25-500 bp)

Lane 2: Y1 (5 μΜ)

Lane 3: Y2 (5 μΜ)

Lane 4: Y3 (5 μΜ)

Lane 5: Y_1-3_DNA (5 μΜ)

Lane 6: Y_1-3_-NS (5 μΜ)

Lane 7: Y_1-3_-NS (5 μΜ) + EpCAM (10 ag/mL)

Lane 8: Y_1-3_-NS (5 μΜ) + EpCAM (1 fg/mL)

**Agarose Gel Electrophoresis of HER2 Analysis.**

Weigh 2.5 g agarose in advance into 100 mL of 1 × TAE solution, mix well, and heat in a microwave oven until the solution is uniform and transparent. Then add 10 μL 4SGeIRed nucleic acid dye to the above mixture, mix well and pour it into the membrane to form 2.5% agarose gel. In electrophoretic assay, each sample channel ended up containing 15 μL sample and 5 μL 6 x Loading buffer. Electrophoresis was performed in a 1 x TAE buffer and energized at 100 V for 70 min. Each lane of the gel represented different samples as detailed in subsequent descriptions.

Figure 4g:

Lane 1: Marker (25-500 bp)

Lane 2: Y4 (5 μΜ)

Lane 3: Y5 (5 μΜ)

Lane 4: Y6 (5 μΜ)

Lane 5: Y_4-6_DNA (5 μΜ)

Lane 6: Y_4-6_-NS (5 μΜ)

Lane 7: Y_4-6_-NS (5 μΜ) + HER2 (10 ag/mL)

Lane 8: Y_4-6_-NS (5 μΜ) + HER2 (1 fg/mL)

**Table S4. Sequences of split G-quadruplex used in the sG4@Ag^+^-nanospheres.**

| Names | Sequences (5'-3') |
| --- | --- |
| sG4a | TAGGGCTTTGGAATATATA |
| sG4a’ | GGGAGGGAGGGAATTTGGACAATAAT |
| sG4b | TAGGGCGGGTTTGGAATATATA |
| sG4b’ | GGGAGGGAGGGAATTTGGACAATAAT |
| sG4c | TAGGGCGGGATTTGGAATATATA |
| sG4c’ | GGGAGGGAATTTGGACAATAAT |
| sG4d | TAGGGCGGGAGGGTTTGGAATATATA |
| sG4d’ | AGGGAATTTGGACAATAAT |

The underlined part is the split G-quadruplex (sG4).

**Table S5. Sequences of split G-quadruplex used in the sG4@Hg^2+^-nanospheres.**

| Names | Sequences (5'-3') |
| --- | --- |
| sG4e | TAGGGCTTTTACTGTAGT |
| sG4e’ | GGGAGGGAGGGAATTTAAGAGTCTG |
| sG4f | TAGGGCGGGTTTTACTGTAGT |
| sG4f’ | GGGAGGGAGGGAATTTAAGAGTCTG |
| sG4g | TAGGGCGGGATTTTACTGTAGT |
| sG4g’ | GGGAGGGAATTTAAGAGTCTG |
| sG4h | TAGGGCGGGAGGGTTTTACTGTAGT |
| sG4h’ | AGGGAATTTAAGAGTCTG |

The underlined part is the split G-quadruplex (sG4).

**Table S6. DNA sequences used in specificity experiments.**

| Names | Sequences (5'-3') |
| --- | --- |
| ssDNA^a^ | ATCCAGATCGTAGTAAC |
| G4 | TAGGGCGGGAGGGAGGGAA |
| ssDNA@Ag^+^-NS | ATCCAGTTTGGAATATATA |
| ssDNA@Ag^+^-NS’ | ATCGTAGTAACTTTGGACAATAAT |
| ssDNA@Hg^2+^-NS | ATCCAGTTTTACTGTAGT |
| ssDNA@Hg^2+^-NS’ | ATCGTAGTAACTTTAAGAGTCTG |

^a^ single-stranded DNA.

**Figure S1**. The chemical structure of [colistin](https://www.chemsrc.com/en/cas/108750-13-6_955452.html).

**Instruments.**

Electrochemical measurements were performed by using a three-electrode system at a CHI-660E Electrochemical Workstation (Shanghai, China). The reference electrode and the auxiliary electrode were the saturated Ag/AgCl and a platinum wire, respectively, and the working electrode was a pre-treated indium tin oxide (ITO) electrode. Differential pulse voltammetry (DPV) was selected for measurement in the range of -0.1~ 0.7 V. The MOPS buffer pH was determined using a PHS-3C Benchtop pH meter (INESA, Shanghai, China). The transmission electron microscopy (TEM) images were obtained using a Tecnai G2F20 STWIN TEM at an accelerating voltage of 200 kV (FEI Co.; USA). The atomic force microscope (AFM) images were performed on Bruker Dimension Icon AFM (Germany). Dynamic Light Scattering (DLS) assay was performed on Malvern Nano ZS90 (Nano ZSP, Malvern, England). a resistivity of 18.25 MΩ · cm. The detection of ^201^Hg and ^107^Ag was performed using an Agilent 8900 ICP-MS (Agilent Technologies, Japan). The 0.22 μm Nylon 66 filter were purchased from Solarbio (Solarbio Co.; Ltd.; Beijing, China). Ultracentrifugation was performed using the Type 32Ti rotor in an Optima XPN-100 ultracentrifuge (Beckman Coulter, Indianapolis, IN, USA). Agarose gel electrophoresis was performed in a submerged gel electrophoresis tank (Sangon Biotech, China), and gel images were recorded with a UV transilluminator gel Doc XR+ (Bio-Rad, USA). Isothermal titration calorimetry (ITC) was conducted with Microcal PEAQ-ITC (Malvern Panalytical, UK). Circular dichroism (CD) analysis was conducted with a BRIGHTTIME Chirascan (AppliedPhotophysics, UK).

**Isothermal Titration Calorimetry**

ITC measurements were recorded at 25 ℃ with a Microcal PEAQ-ITC isothermal titration calorimeter (Malvern Panalytical, UK). Colistin solutions (60 µL, 40 µM) in the syringe were titrated into the sample cell filled with G4 (260 µL, 500 µM) with 150 s intervals between each injection. Experiments were designed to use an initial injection of 0.4 µL followed by 19 injections of 2.0 µL. The data were analyzed with the Microcal Origin software using one set of binding site models.

**Figure S2.** ITC analysis of the interaction between non-G4 single-stranded DNA (ssDNA) and colistin. (a) Raw thermogram obtained by titrating colistin into the ssDNA solution. (b) Integrated heat changes plotted against the molar ratio.

**Figure S3.** UV-vis absorption spectra of G4 under different incubation systems. (a) G4 mixed with EpCAM and HER2 proteins; (b) G4 co-incubated with colistin and Ag^+^; (c) G4 co-incubated with colistin and Hg^2+^; (d) G4 mixed with colistin and plasma. The elevated baseline absorbance from 300 to 600 nm in panels (b-d) indicates colistin-triggered G4 aggregation.

**Figure S4.** Circular dichroism spectra of sG4@Ag^+^-NS (a) and sG4@Hg^2+^-NS (b).

**Cell Recovery.** Cryopreserved human breast cancer SKBR-3 and MCF-7 cells were retrieved from liquid nitrogen and immediately transferred to a sterile 37 °C water bath for thawing. To prevent contamination, the cryovial was placed inside a sterile sealed PE glove, ensuring the tube opening remained above the water level. During the thawing process, the cryovial was shaken rapidly and evenly to facilitate complete cell thawing. The thawed cell suspension was transferred to a sterile Eppendorf tube, thoroughly resuspended in fresh McCoy's 5S medium supplemented with 10% fetal FBS of SKBR-3 cells and DMEM high glucose medium supplemented with 10% fetal FBS of MCF-7 cells, and then centrifuged at 1000 rpm for 3 minutes. After centrifugation, the supernatant was carefully aspirated and discarded. The cell pellet was gently resuspended in fresh medium by slow pipetting; following thorough mixing, the cell suspension was transferred to a sterile culture dish pre-filled with fresh medium. The culture dish was gently swirled horizontally to ensure uniform cell distribution, and finally incubated in a cell culture incubator at 37 °C.

**Cell Culture, Passage and sEVs Isolation.** The breast cancer cells were cultured in fresh medium supplemented with 10% FBS and incubated at 37 °C with 5% CO_2_. Cell passage was performed when cell confluency reached 70%-80% and the cells were in the logarithmic growth phase. Prior to passage, cell viability was confirmed by microscopic observation. Experimental supplies including sterile culture dishes, Eppendorf tubes, sterile pipette tips, and Pasteur pipettes were prepared. Cell culture medium, PBS, and 0.25% trypsin were pre-warmed to room temperature in advance. For adherent human breast cancer cells, the original medium was first aspirated slowly, and the cells were gently rinsed 3 times with sterile PBS. After discarding PBS, an appropriate amount of 0.25% trypsin was added to the culture dish to ensure uniform coverage of the cell surface. The culture dish was gently swirled to facilitate full contact between trypsin and cells, followed by microscopic observation. When most cells became rounded, shrank in size, and intercellular spaces enlarged, trypsin was immediately aspirated, and serum-containing complete medium was added to terminate digestion. The cell surface was gently yet thoroughly pipetted to detach all digested cells. The cell suspension was collected into a sterile Eppendorf tube and centrifuged at 1000 rpm for 5 minutes in a horizontal centrifuge. After centrifugation, the supernatant was carefully aspirated, and an appropriate volume of fresh medium was added to resuspend the cell pellet. The cell suspension was repeatedly pipetted to ensure complete dispersion and homogenization. A 20 µL aliquot of the cell suspension was slowly added to the edge of the coverslip on a hemocytometer, allowing it to fill the counting chamber via capillary action. The hemocytometer was left undisturbed for several minutes to allow complete cell sedimentation, after which cell counting was performed under a microscope.

After incubation, all cell culture supernatants were collected into 50-mL sterile conical tubes for gradient centrifugation to extract sEVs. As shown in Scheme S1a, the collected conditioned medium was initially centrifuged at 500 g for 10 minutes at 4 °C to pellet cells and cell debris. The supernatant was then centrifuged at 2,000 g for 20 minutes at 4 °C to further remove cell debris and apoptotic bodies. The resulting supernatant was centrifuged at 10,000 g for 30 minutes at 4 °C to remove large extracellular vesicles. The supernatant from the previous step was filtered through a 0.22 μm polyethersulfone filter to minimize microparticle contamination. The filtered medium underwent ultracentrifugation at 150,000 g for 2 hours at 4 °C. The resultant pellet was washed with PBS and recentrifuged under the same conditions. Finally, the isolated sEVs were resuspended in 200 μL PBS and stored at -80 °C until further use. The sEVs were characterized using nanoparticle tracking analysis (NTA), transmission electron microscopy, and western blotting.

**Scheme S1**. Workflow schematic for the isolation and enrichment of sEVs from (a) Cell culture supernatant, and (b) clinical specimens.

**sEVs Isolation of Clinical Plasma Samples.**

The whole blood samples collected were immediately centrifuged within 2 hours of collection at 2,500 g for 15 minutes at 25 °C to pellet the red blood cells, white blood cells, and platelets (Scheme S1b). The plasma was then centrifuged under the same conditions to isolate platelet-poor plasma. The resulting plasma samples were diluted 1:20 in ice-cold PBS. Following this, a further centrifugation step at 10,000 g for 30 minutes at 4 °C was performed. The plasma, thus pretreated, was then passed through a 0.22 μm syringe filter, a process designed to eliminate large vesicles, cell debris, and other impurities. The filtered sample was ready for subsequent ultracentrifugation procedures. Further steps and protocols are detailed in the “**Cell Culture, Passage and sEVs Isolation**” section.

**Nanoparticle Tracking Analysis (NTA).**

The particle concentration and size distribution of sEVs isolated from SKBR-3 cells and human plasma samples were analyzed using the calibrated Zeta View (Particle Metrix, Meerbusch, Germany). Each sample was diluted 10000-fold in particle-free PBS before manual introduction. Instrumental parameters were set at a cell temperature of 24 °C, sensitivity 70, shutter 70, Max Area 1000, Min Area 5, and Min Brightness 20. Nanoparticle Tracking Analysis Software (ZNTA) v 8.05.04 was used for data capture and analysis, averaging three replicates for each reported value.

**Western Blotting.**

Proteins were extracted from sEVs using modified RIPA lysis buffer (50 mM Tris pH 6.6, 1 mmol/L EDTA, 150 mM NaCl, 0.1% SDS, 1% sodium deoxycholate, 1% Triton X-100) supplemented with protease inhibitors on ice. Protein concentration was quantified using a BCA assay kit. Protein solutions and working reagents were prepared and incubated at 37 °C for 30 minutes, followed by measurement of optical density values at 595 nm using a microplate reader. A protein concentration standard curve was generated to calculate the extracted protein concentration. sEVs proteins were mixed with 5×Loading buffer (25% of the original protein volume), vortexed thoroughly, and denatured in a metal bath at 95 °C for 10 minutes. Equal amounts of protein were separated by SDS-PAGE electrophoresis and transferred to Immobilon-FL PVDF membranes (Merck Millipore Ltd). The membranes were blocked with 5% BSA for 1 hour and subsequently incubated overnight with the following primary antibodies: anti-CD63 antibody (Proteintech, Rosemont, IL, USA), 1:1000; anti-CD81 antibody (Proteintech, Rosemont, IL, USA), 1:1000; anti-CD9 antibody (Proteintech, Rosemont, IL, USA), 1:1000; anti-EpCAM antibody (Thermo Fisher Scientific, Waltham, MA, USA), 1:1000; anti-HER2 antibody (Proteintech, Rosemont, IL, USA), 1:1000; anti-Apolipoprotein B (Proteintech, Rosemont, IL, USA), 1:1000. The next day, membranes were incubated with IRDye 680-conjugated goat anti-rabbit IgG (H+L) (LI-COR Biosciences, NE, USA) at room temperature for 1 hour. Fluorescent signals were visualized using an Odyssey imaging system (LI-COR, Bad Homburg, Germany).

**Table S7.** Operational Parameters of ICP-MS

| Parameter | Value |
| --- | --- |
| Radiofrequency power | 1550 W |
| Coolant argon gas flow rate | 1 mL/min |
| Carrier (nebulizer) gas flow rate | 15 mL/min |
| Auxiliary argon gas flow rate | 1 mL/min |
| Scanning mode | Peak area |
| Ingestion time | 25 s |
| Dwelling time | 30 s |
| Isotope monitored | ^201^Hg; ^107^Ag |

**Figure S5**. Specific binding between target protein and nanospheres. (a) Ag^+^-NS (EpCAM); (b) Hg^2+^-NS (HER2). Triple measurements calculated error bars and represented standard deviations.

**Optimization of EpCAM and HER2 Analysis Conditions.** To achieve superior analytical performance, experimental conditions were optimized using EpCAM and HER2 as markers. The self-assembly conditions of the nanospheres were prioritized for optimization. For the sG4@Ag⁺-NS system, the maximum signal difference was observed when the concentrations of DNA strands were set to 40 μM (Figure S6a and b). The signal difference reached its optimum value when the Ag^+^ concentration was 600 μM (Figure S6c and d). When 80 mM was chosen as the concentration of KNO_3_, a relatively large difference in electrochemical signals could be obtained (Figure S6e and f). Accordingly, it was better to control the self-assembly time of the nanospheres at room temperature after annealing for 3 h (Figure S6g and h).

**Figure S6**. Optimization of conditions based on self-assembly of Ag⁺-NS. (a-b) The concentration of Y1-Y3 and sG4a; (c-d) The initial concentration of Ag^+^; (e-f) The initial concentration of K^+^; (g-h) Ag^+^-NS self-assembly time. Triple measurements calculated error bars and represented standard deviations.

For sG4@Hg^2+^-NS system, the maximum signal difference was observed when the concentrations of DNA strands were set to 20 μM (Figure S7a and b). The highest signal-to-noise ratio observed at 600 μM for Hg^2+^ concentration (Figure S7c and d). When 60 mM was chosen as the concentration of KNO_3_, a relatively large difference in electrochemical signals could be obtained (Figure S7e and f). The optimal incubation time for Hg^2+^-NS self-assembly was established at 3 h (Figure S7g and h).

**Figure S7**. Optimization of conditions based on self-assembly of Hg^2^⁺-NS. (a-b) The concentration of Y4-Y6 and sG4a; (c-d) The initial concentration of Hg^2^⁺; (e-f) The initial concentration of K^+^; (g-h) Hg^2^⁺-NS self-assembly time. Triple measurements calculated error bars and represented standard deviations.

Subsequently, the analytical conditions were systematically optimized. The maximum signal difference was achieved when the volume of added sG4@Ag⁺-NS was adjusted to 20 μL (Figure S8a and b). The signal difference reached its optimum value when the sG4@Hg^2^⁺-NS volume was 15 μL (Figure S8c and d). Accordingly, after 60 minutes of reaction incubation, the electrical signals of the two nanospheres tended to stabilize, indicating that the reaction had proceeded sufficiently (Figure S8e and f). When the concentration of colistin was 20 μM, the signal difference for EpCAM and HER2 was maximum (Figure S8g-j), and when the added colistin time was 30 minutes, which achieved the optimal signal difference (Figure S8k and l).

**Figure S8.** Optimization of EpCAM and HER2 analysis conditions. The volume of Ag⁺-NS (a and b) and Hg^2^⁺-NS (c and d); (e and f) the reaction time of protein and nanospheres; (g-j) the concentration of colistin; (k and l) The time of adding colistin. Triple measurements calculated error bars and represented standard deviations.

**Figure S9.** The DPV response of different concentration EpCAM and HER2 (a) without colistin and (b) with colistin.

**Figure S10**. Time-dependent kinetic electrochemical curves to verify the sequential sensing process of Ag^+^-NS (a) and Hg^2+^-NS (b). Target proteins (EpCAM for Ag^+^-NS, HER2 for Hg^2+^-NS) were incubated with three types of nanospheres (nanospheres, ssDNA-modified nanospheres, sG4-functionalized nanospheres) within the first 60 min, and colistin was supplemented at the 60 min time point (green arrow). Triple measurements calculated error bars and represented standard deviations.

**Figure S11.** The inter-day repeatability (a), intra-day repeatability (b) and inter-batch repeatability (c) of the DNA nanospheres. Triple measurements calculated error bars and represented standard deviations.

**Figure S12.** Evaluation of cross-reactivity and interference in (a) EpCAM positive and (b) HER2 positive SKBR3-sEVs detection. All sEVs samples were diluted to 1×10^6^ particles/mL. The concentrations of the interfering proteins and ssDNA were 1 pg/mL and 5 μM, respectively. ssDNA: single-stranded DNA. Triple measurements calculated error bars and represented standard deviations.

**Table S8.** Calibration uncertainty of the calibration curve.

| Target | Adjusted *R*^2^ | Slope ± SE | Slope 95% CI | Intercept ± SE | Intercept 95% CI |
| --- | --- | --- | --- | --- | --- |
| EpCAM protein without colistin | 0.9587 | 76.35 ± 6.80 | 54.72-97.98 | 565.11 ± 15.98 | 514.27-615.95 |
| HER2 protein without colistin | 0.9496 | 53.89 ± 6.27 | 33.95-73.83 | 374.09 ± 16.07 | 322.95-425.23 |
| EpCAM protein with colistin | 0.9707 | 100.70 ± 8.72 | 72.97-128.43 | 263.25 ± 23.47 | 188.57-337.93 |
| HER2 protein with colistin | 0.9753 | 67.80 ± 5.38 | 50.67-84.93 | 202.30 ± 14.49 | 156.18-248.42 |
| EpCAM-sEVs with colistin | 0.9809 | 71.74 ± 4.47 | 59.35-84.14 | 209.99 ± 21.49 | 150.32-269.66 |
| HER2-sEVs with colistin | 0.9793 | 58.10 ± 4.22 | 44.69-71.51 | 183.00 ± 17.88 | 126.10-239.90 |

*R*^2^: coefficient of determination; SE: standard error; 95% CI: 95% confidence interval.

**Table S9.** Noise-floor analysis of the electrochemical sensing system.

| Target | n | Blank current, mean ± SD | RSD (%) | Noise-floor threshold |
| --- | --- | --- | --- | --- |
| Ag^+^-NS system | 11 | 643.1 ± 17.8 | 2.77 | 696.5 |
| Hg^2+^-NS system | 11 | 439.4 ± 13.0 | 2.96 | 478.4 |

The noise floor was calculated from 11 repeated measurements of no-target blank samples. The threshold was defined as the blank mean plus three times the standard deviation. ^a^RSD = standard deviation / arithmetic mean of calculated results (X) × 100%.

**Table S10.** Relative standard deviation (RSD) values of long-term reproducibility.

| Test number |  | 1 | 2 | 3 | 4 | 5 | 6 | 7 | 8 | 9 | 10 | 11 |
| --- | --- | --- | --- | --- | --- | --- | --- | --- | --- | --- | --- | --- |
|  | sG4@Ag^+^-NS | 580 | 577 | 586 | 545 | 583 | 571 | 582 | 555 | 569 | 596 | 571 |
|  | sG4@Hg^2+^-NS | 417 | 407 | 413 | 402 | 421 | 418 | 428 | 431 | 409 | 427 | 398 |
| Mean value | sG4@Ag^+^-NS | 574 | | | | | | | | | | |
|  | sG4@Hg^2+^-NS | 416 | | | | | | | | | | |
| Standard  Deviation | sG4@Ag^+^-NS | 14.4 | | | | | | | | | | |
|  | sG4@Hg^2+^-NS | 10.8 | | | | | | | | | | |
| RSD^a^ | sG4@Ag^+^-NS | 2.50% | | | | | | | | | | |
|  | sG4@Hg^2+^-NS | 2.61% | | | | | | | | | | |

After storage for 7 days, sG4@Ag^+^-NS was incubated with 100 ag/mL EpCAM, and sG4@Hg^2+^-NS was incubated with 100 ag/mL HER2. Eleven parallel reaction tubes were tested for each nanosphere system to evaluate the reproducibility of the electrochemical responses. ^a^RSD = standard deviation / arithmetic mean of calculated results (X) × 100%.

**Plasma stability and spiked recovery experiments** Healthy human plasma was serially diluted to different fold concentrations with MOPS. Subsequently, sG4@Ag^+^-NS or sG4@Hg^2+^-NS, and colistin were added to the pre-diluted plasma. After incubation for 30 minutes, electrochemical signals were detected. Finally, it was found that the signal of the nanospheres was no longer interfered with when the plasma was diluted 10,000-fold. We further conducted spiked recovery experiments at this concentration, and the detailed procedure was illustrated in Scheme S2.

**Scheme S2**. Workflow schematic for the spiked recovery experiments.

**The Calculation of Limit of Detection (LOD)^[1]^**

According to the literature, the LOD was calculated by giving an electrochemistry signal three times standard deviation (SD) lower than the background signal in the absence of a target.

First, the electrochemistry signal-target concentration curve obtained by least-square root linear regression was the standard curve. The standard curve of was plotted as:

***Y* = 𝐴 Log*C* + *B*	(1)**

Where A and B were the slope and intercept of the standard curve, respectively. *C* was the concentration of target, and Y represented the corresponding electrochemistry value.

When:

**𝑌 = 𝑌_blank_ - 3𝑆𝐷	(2)**

Where SD was the standard deviation and *Y*_blank_ was the electrochemistry signal of the blank sample (without target).

The LOD was calculated as:

***LOD* = 10 ^[( 𝑌^_blank_ ^- 3^*^SD^* ^) –^ *^B^*^] /^ *^A^*	(3)**

**Table S11.** Comparison of methods for the determination of EpCAM and HER2

| Method | Target | System | Time (min) | Homogeneous | Linear range; LOD^j^ (/mL) | Verify practicality | Purpose | Reference |
| --- | --- | --- | --- | --- | --- | --- | --- | --- |
| EC^a^ | EpCAM | QZIFh-67/Au/MB | 13 | Yes | 5 pg-100 ng;  3 pg | Cell line | Therapeutic drug monitoring | ^[2]^ |
| EC | EpCAM | Au NPs; aptamer; SnO₂; APTES | 30 | No | 20 pg-500 ng; 24.4 pg | Not mentioned | Not mentioned | ^[3]^ |
| EC | EpCAM | DNA nanohydrogel; RCA; Aptamer; Pb^2+^ | 45 | Yes | 100 ag-10 fg; 30 ag | Human plasma samples | Diagnosis and staging | ^[4]^ |
| PEC^b^ | EpCAM | In_2_S_3_@BiOBr_0.8_I_0.2_/PEDOT; AuNPs | 150 | No | 0.1-100 ng;  0.996 pg | Spiked human serum samples | Not mentioned | ^[5]^ |
| EC | EpCAM | CuS@PEDOT:PSS/WP | < 10 | No | 10 fg-1000 ng;  10 fg | Spiked human serum samples | Not mentioned | ^[6]^ |
| EC | EpCAM | Co-Fe-MOF nanomaterial | Not mentioned | Yes | 0-20 pg;  8.4 fg | Spiked human serum samples | Not mentioned | ^[7]^ |
| ECL^c^ | EpCAM | SiO_2_; magnetized nickel | 360 | No | 15 pg-420 pg;  5.5 pg | Not mentioned | Not mentioned | ^[8]^ |
| SERS^d^ | EpCAM | GO-Au NP | 480 | Yes | 8.47 pg-74.78 pg;  3.63 pg | Cell line | Not mentioned | ^[9]^ |
| Colorimetry | EpCAM | Aptamer/CoFe-350j/Pt/ Fe_3_O_4_/PDDA/Au/TMB-H_2_O_2_ | 70 | No | 1 pg-100 ng;  0.89 pg | Cell line | Not mentioned | ^[10]^ |
| SERS | EpCAM | Antibody/Fe_2_O_3_@Ag/magnetic nanoparticles | 120 | No | 1 pg-15 pg; 5 pg | Spiked human serum samples | Not mentioned | ^[11]^ |
| ICP-MS^e^ | EpCAM | Aptamer/AuNPs/UCNP(Tb) | 40 | No | 500 ng-6 μg;  74 ng | Human plasma samples | Diagnosis | ^[12]^ |
| EC | EpCAM | DNA nanomachine; HCR; aptamer | 60 | Yes | 10 ag-100 fg; 3 ag | Human plasma samples | Diagnosis and staging | ^[13]^ |
| Colorimetry | HER2 | MOFs; RCA; aptamer; streptavidin; biotin | 70 | No | 12 fg-1.2 pg; 12 fg | Human serum samples | Diagnosis and subtypes | ^[14]^ |
| LFIA^f^ | HER2 | AuNP; antibody; PVC; NC membrane | <15 | No | 1.7 ng-400 ng; 1.7 ng | Spiked human serum samples | Not mentioned | ^[15]^ |
| ECL | HER2 | ZMB/Au@Pt/HBA; Aptamer; Zr-MOF | 60 | No | 10 fg-100 ng; 0.2 fg | Spiked human serum samples | Not mentioned | ^[16]^ |
| ECL | HER2 | ALP; ascorbic acid; Co_9_S_8_@ZnIn_2_S_4_; biotin antibody | 270 | No | 10 pg-10 ng; 3.5 pg | Human serum samples | Not mentioned | ^[17]^ |
| EC | HER2 | Aptamer; Co-UiO-66; ferrocene; toluidine blue | 40 | No | 100 fg-10 ng;  10 fg | Human serum samples | Diagnosis | ^[18]^ |
| EC | HER2 | Antibody; hydrogel; polymer poly(3,4-ethylenedioxythiophene); peptide | 60 | No | 100 pg-1000 ng;  45 pg | Human serum samples | Not mentioned | ^[19]^ |
| Fluorescence | HER2 | Aptamer; MNAzyme; CHA; QDs; CDs | 200 | Yes | 10 ag-10 fg;  3 ag | Human serum samples | Diagnosis | ^[20]^ |
| EC | HER2 | FeMn-NCₑₜcₕ/SAC; TMB; ZnO@ZIF-8 | 140 | No | 10 pg-10 ng;  3.9 pg | Human serum samples | Not mentioned | ^[21]^ |
| Colorimetry | HER2 | Zr-MOF; AuNPs; Apt-bio; TMB; nanozyme | 90 | No | 100 fg-150 ng;  30.226 fg | Human serum samples | Diagnosis | ^[22]^ |
| SPRM^g^ | EpCAM & HER2 | Aptamer; HRP-streptavidin; tetramethylbenzidine | 180 | No | Not mentioned | Human plasma samples | Diagnosis | ^[23]^ |
| SCE^h^ | EpCAM & HER2 | Nanosphere; microfluidic; QDs; fluorescent tag | 90 | No | Not mentioned | Cell line | Subtypes | ^[24]^ |
| EC | EpCAM & HER2 | Zr-MOF@MB; antibody; mAuNSs; nanostars | 25 | No | Not mentioned | Human plasma samples | Diagnosis | ^[25]^ |
| ddPCR^i^ | EpCAM & HER2 | Antibody-DNA conjugates; immuno-PCR; biotin; FAM; Cy5 | 240 | No | Not mentioned | Human plasma samples | Subtypes | ^[26]^ |
| EC | EpCAM & HER2 | DNA nanomachine; G-quadruplex; colistin; Ag⁺; Hg^2+^ | 90 | Yes | EpCAM: 1 ag-1 fg; 0.5 ag  HER2: 1 ag-1 fg; 0.8 ag | Human serum samples | Diagnosis and subtypes | This work |

^a^: Electrochemistry; ^b^: Photoelectrochemical; ^c^: electrochemiluminescence; ^d^: Surface-enhanced Raman scattering; ^e^: Inductively coupled plasma-Mass Spectrometry; ^f^: Lateral flow immunoassay; ^g^: Surface plasmon resonance microscopy; ^h^: Spectrally combined encoding; ^i^: Droplet digital immuno-PCR; ^j^: Limit of detection.

**Table S12.** Determination of EpCAM-sEVs in spiked plasma samples (n = 3).

| Sample | Added (particles/mL) | Found (particles/mL) | Recovery (%) | RSD^a^ (%) |
| --- | --- | --- | --- | --- |
| 1 | 10^3^ | 1.06 × 10^3^ ± 25 | 106.3 | 2.36 |
|  | 10^4^ | 1.03 × 10^4^ ± 218 | 103.4 | 2.12 |
|  | 10^5^ | 9.78 × 10^4^ ± 1866 | 97.8 | 1.91 |
| 2 | 10^3^ | 1.02 × 10^3^ ± 20 | 102 | 1.96 |
|  | 10^4^ | 9.93 × 10^3^ ± 195 | 99.3 | 1.96 |
|  | 10^5^ | 9.95 × 10^4^ ± 2298 | 99.5 | 2.31 |
| 3 | 10^3^ | 1.04 × 10^3^ ± 27 | 103.7 | 2.60 |
|  | 10^4^ | 1.03 × 10^4^ ± 236 | 102.8 | 2.29 |
|  | 10^5^ | 1.05 × 10^5^ ± 1869 | 105.0 | 1.78 |

^a^RSD = standard deviation / arithmetic mean of calculated results (X) × 100%.

**Table S13.** Determination of HER2-sEVs in spiked plasma samples (n = 3).

| Sample | Added (particles/mL) | Found (particles/mL) | Recovery (%) | RSD^a^ (%) |
| --- | --- | --- | --- | --- |
| 1 | 10^3^ | 1.01 × 10^3^ ± 20 | 101.1 | 1.98 |
|  | 10^4^ | 9.42 × 10^3^ ± 241 | 94.2 | 2.56 |
|  | 10^5^ | 9.97 × 10^4^ ± 1814 | 99.7 | 1.82 |
| 2 | 10^3^ | 1.06 × 10^3^ ± 25 | 106.2 | 2.36 |
|  | 10^4^ | 1.01 × 10^4^ ± 218 | 100.8 | 2.16 |
|  | 10^5^ | 1.03 × 10^5^ ± 2019 | 103.0 | 1.96 |
| 3 | 10^3^ | 1.01 × 10^3^ ± 26 | 101.3 | 2.57 |
|  | 10^4^ | 1.05 × 10^4^ ± 240 | 105.2 | 2.29 |
|  | 10^5^ | 9.86 × 10^4^ ± 2320 | 98.6 | 2.35 |

^a^RSD = standard deviation / arithmetic mean of calculated results (X) × 100%.

**Table S14.** Comparison of various methods for detecting tumor-derived exosomes

| Methods | System | Time (min)^a^ | Linear range; LOD^b^ (particles/mL) | Verify practicality | Specificity in complex matrices | Purpose | Reference |
| --- | --- | --- | --- | --- | --- | --- | --- |
| ExoView | Antibody microarray; compared with NTA, MRPS | 120 | 1 × 10^7^-1 × 10^9^; Not mentioned | Human cell lines | Not mentioned | Single-vesicle characterization | ^[27]^ |
| NanoFCM | Aptamer labeling; nFCM; FAM fluorophore | Within hours | Not mentioned | Human plasma samples (n = 69) | Plasma sEVs | Diagnosis and Subtypes | ^[28]^ |
| Microfluidic system | 3D herringbone nanopatterned microfluidic chip; antibody capture; fluorescence | 75 | Not mentioned; 1 × 10^4^ | Human plasma samples (n = 20) | sEVs subpopulations in plasma | Diagnosis | ^[29]^ |
| Digital ELISA | Magnetic beads; Antibody; HRP | Not mentioned | Not mentioned; 9 × 10^3^ | Plasma-compatible digital EV detection | Plasma sEVs | Single-EV detection | ^[30]^ |
| CRISPR | LbaCas12a; LbuCas13a; gRNA; | 90 | Not mentioned; 2.14 × 10^5^ | Human plasma samples (n = 31) | Plasma sEVs | Diagnosis | ^[31]^ |
| Single-EV assay | GRP; Cy3; Aptamer; drFRET | 70 | 1 × 10^5^-1 × 10^9^; 7.7 × 10^4^ | Human serum sample (n = 100) | Serum sEVs | Diagnosis | ^[32]^ |
| Plasmonic technology | Antibody; HRP; Plasmonic nanoring resonator | 50 | Not mentioned; 1 × 10^3^ | Human plasma samples (n = 106) | Plasma sEVs | Treatment response | ^[33]^ |
| Nanopore sensing | Aptamer; SA-MBs; DNA scaffold; TDN | 120 | 1 × 10^5^-1 × 10^7^; 1 × 10^5^ | Human plasma samples (n = 35) | Plasma sEVs | Diagnosis and Staging | ^[34]^ |
| Fluorescence | Thermophoresis; Cy5/BHQ3; Aptamer; Microbead | 150 | 2 × 10^6^-2 × 10^9^; 2.8 × 10^5^ | Human serum sample (n = 30) | Serum sEVs | Diagnosis and Subtypes | ^[35]^ |
| Fluorescence | CRISPR-Cas12a; DNA; Antibody; | 30 | Not mentioned; 2 × 10^3^ | Human plasma samples (n = 31) | Plasma sEVs | Diagnosis | ^[31]^ |
| Fluorescence | DNA nanomotor; AuNP; AFM | 180 | 1.56×10^9^- 1×10^11^; 6.01 × 10^8^ | Human serum sample (n = 63) | Serum sEVs | Diagnosis | ^[36]^ |
| Fluorescence | DNA; PEG; thermophoretic; Cy3; Cy5 | 15 | 5×10^9^-1×10^11^; 1.03 × 10^8^ | Human serum sample (n = 33) | Serum sEVs | Diagnosis | ^[37]^ |
| Fluorescence | Aptamer; split  G-quadruplex; thioflavin T | 90 | 1×10^3^-1×10^9^; 155 | Human plasma samples (n = 127) | Plasma sEVs | Diagnosis and Subtypes | ^[38]^ |
| Colorimetry | Biotin; HRP; Streptavidin; DNA | 120 | 5.0 × 10^6^-10^9^; 1 × 10^6^ | Not mentioned | Not mentioned | Not mentioned | ^[39]^ |
| Colorimetry | PDVB; SiO_2_; Biotin; Streptavidin | 90 | 2.3 × 10^5^-10^10^; 1.8 × 10^5^ | Human plasma samples (n = 60) | Plasma sEVs | Diagnosis | ^[40]^ |
| SERS^c^ | AuNPs; DNA; Biotin; Exo I; Streptavidin;Beads | 180 | Not mentioned; 2 × 10^4^ | Human plasma samples (n = 30) | Plasma sEVs | Diagnosis | ^[41]^ |
| SERS | AuSts; p-NTP; SiO_2_; Aptamer | Not mentioned | 1.0 × 10^8^-3.0 × 10^12^; 1.0 × 10^7^ | Human serum sample (n = 26) | Serum sEVs | Diagnosis and Subtypes | ^[42]^ |
| Flow cytometry | Agarose microgel; RCA; Antibody; size exclusion chromatography | 180 | Not mentioned; 5 × 10^3^ | Human plasma samples (n = 15) | Plasma sEVs | Diagnosis | ^[43]^ |
| SPRM^d^ | Aptamers; 5-T spacer; ligand; sensor chips | 180 | 1.0 × 10^6^-10^9^; 4.6 × 10^6^ | Human plasma samples (n = 85) | Plasma sEVs | Diagnosis | ^[23]^ |
| EC^e^ | DNA; MB; GB@PTB; N_3_ | < 60 | 2.5 × 10^3^-2.5 × 10^8^; 325 | Human plasma samples (n = 111) | Plasma sEVs | Diagnosis | ^[44]^ |
| EC | alkaline phosphatase; Antibody; Biotin; Streptavidin | > 180 | 1.0 × 10^3^-1.0 × 10^6;^ 1.0 × 10^3^ | Not mentioned | Not mentioned | Not mentioned | ^[45]^ |
| Immunomagnetic capture | SPE; Immunomagnetic bead; Biotin; Antibodies; SA-HRP; TMB | < 60 | Not mentioned; 1.0 × 10^3^ | Human plasma samples (n = 142) | Plasma sEVs | Diagnosis and monitoring | ^[46]^ |
| EC | CRISPR-Cas12a; DNA; MB | 300 | 7 × 10^2^-1 × 10^6^; 220 | Human serum sample (n = 17) | Serum sEVs | Diagnosis and Subtypes | ^[47]^ |
| EC | MOFs; MB; G-quadruplex | 80 | 1.7 × 10^1^-3.4 × 10^5^; 5 | Not mentioned | Not mentioned | Not mentioned | ^[48]^ |
| EC | DNA nanomachine; G-quadruplex; colistin; Ag⁺; Hg^2+^ | 90 | EpCAM:10^2^-1 × 10^7^; 30  HER2:10^2^-1 × 10^6^; 50 | Human plasma samples (n = 85) | Plasma sEVs | Diagnosis and Subtypes | This work |

^a^: Excluding sEVs isolation and enrichment; ^b^: Limit of Detection; ^c^: Surface-enhanced Raman scattering; ^d^: Surface plasmon resonance microscopy; ^e^: Electrochemistry.

**Table S15.** Comparison of commercial and routine workflows for sEVs-related analysis.

| Methods | Target multiplexing | Turnaround time | Complexity | Throughput | Public cost | Workflow integration | URL |
| --- | --- | --- | --- | --- | --- | --- | --- |
| PS Capture sEVs ELISA Kit | PS-positive EVs | 3-5 h | Medium | 96-well format | $549/96 tests | Commercial kit; suitable for EV quantification, but requires capture and washing | <https://labchem-wako.fujifilm.com/us/product/detail/W01W0129-8060.html> |
| ExoELISA-ULTRA CD63, SBI | Single-EV | 4-6 h | Medium | 96 reactions | $1102/96 reactions | Commercial EV marker assay; single-marker readout | <https://www.systembio.com/exoelisa-ultra-complete-kit-cd63-detection> |
| EXOCET, SBI | Total EV quantification | 1-2 h | Low-medium | 96 reactions | $802/96 reactions | Rapid total EV quantification; lacks molecular marker specificity | <https://www.systembio.com/exocet-sEVs-quantitation-kit> |
| Exo-Check Antibody Array, SBI | 8 EV markers | 1 day | High | 8 arrys | $1244/8 arrays | EV marker profiling; not designed for rapid clinical testing | <https://www.systembio.com/products/exosome-research/exosome-detection/arrays/exo-check-exosome-antibody-array> |
| ExoView R100 service, CHLA | Single-EV | 1-2days | High | Service-dependent | $197/test | Single-vesicle phenotyping; requires dedicated platform and analysis | <https://chatgpt.com/g/g-p-6a4c54ae01b48191af2ad0d42773f1cd/c/6a4c559d-b070-83e8-90ee-8f396ca1bcaf> |
| CellStream nano-flow service, CHLA | Single-particle analysis | Service-dependent | High | Service-dependent | $75-150/h | Single-particle analysis; useful for EV characterization, but instrument-dependent | <https://www.chla.org/research/core/extracellular-vesicle-and-nanoparticle-analytics-ev-np/fee-structure> |
| NTA | Total concentration | 0.5-1 h | Medium | Low | About $20/h | Useful for EV QC and particle quantification; lacks molecular marker specificity | / |
| Western blotting | EV markers or target proteins; low multiplexing | 1-2 days | High | Low | Antibody/instrument-dependent | Standard EV characterization method; semi-quantitative and not rapid | / |
| Bead-based flow cytometry | Multiple EV surface markers | 4-6 h | Medium-High | Moderate | Kit/instrument-dependent | Useful for multiplex EV phenotyping; requires antibody panels and cytometry workflow | / |
| This work | Dual-marker sEVs | 1.5 h | Low-Medium | Moderate-High | < $3/test | Antibody-free, enzyme-free, and no electrode modification | This work |

**Table S16.** Information of clinical samples used for comparison between ultracentrifugation and filtration methods.

| No. | Age | Sex | Clinical diagnosis | HER2 status | With colistin | | | | Without colistin | | | |
| --- | --- | --- | --- | --- | --- | --- | --- | --- | --- | --- | --- | --- |
|  |  |  |  |  | EpCAM | | HER2 | | EpCAM | | HER2 | |
|  |  |  |  |  | UC | Filter | UC | Filter | UC | Filter | UC | Filter |
| 1 | 43 | female | Healthy | / | 441 | 464 | / | / | 455 | 400 | / | / |
| 2 | 31 | female | Healthy | / | 425 | 469 | / | / | 515 | 620 | / | / |
| 3 | 35 | male | Healthy | / | 452 | 405 | / | / | 527 | 795 | / | / |
| 4 | 51 | female | Healthy | / | 423 | 383 | / | / | 539 | 513 | / | / |
| 5 | 27 | male | Healthy | / | 453 | 432 | / | / | 560 | 517 | / | / |
| 6 | 47 | female | Healthy | / | 501 | 442 | / | / | 480 | 580 | / | / |
| 7 | 35 | female | Breast cancer | Positive | 647 | 645 | 316 | 387 | 515 | 685 | 783 | 684 |
| 8 | 81 | female | Breast cancer | Positive | 598 | 462 | 324 | 304 | 532 | 745 | 595 | 443 |
| 9 | 68 | female | Breast cancer | Negative | 602 | 588 | 266 | 263 | 642 | 735 | 465 | 498 |
| 10 | 36 | female | Breast cancer | Negative | 513 | 543 | 223 | 237 | 683 | 632 | 432 | 483 |
| 11 | 45 | female | Breast cancer | Negative | 665 | 687 | 230 | 256 | 745 | 780 | 404 | 385 |
| 12 | 46 | female | Breast cancer | Negative | 523 | 491 | 252 | 271 | 796 | 678 | 427 | 449 |
| 13 | 64 | female | Breast cancer | Positive | 452 | 405 | 345 | 389 | 888 | 952 | 413 | 458 |
| 14 | 48 | female | Breast cancer | Negative | 677 | 708 | 272 | 245 | 567 | 488 | 577 | 465 |
| 15 | 39 | female | Breast cancer | Positive | 649 | 580 | 423 | 431 | 622 | 570 | 656 | 797 |
| 16 | 44 | female | Breast cancer | Positive | 892 | 677 | 397 | 308 | 531 | 718 | 703 | 488 |
| 17 | 33 | female | Breast cancer | Positive | 663 | 643 | 260 | 233 | 658 | 581 | 779 | 878 |
| 18 | 52 | female | Breast cancer | Negative | 637 | 664 | 258 | 281 | 807 | 728 | 577 | 465 |

All electrochemical signal values presented in this table represent the mean of three

independent replicate measurements.

**Table S17.** Clinical diagnosis and electrochemical test results of clinical cancer patients

| No. | Age | Clinical diagnosis | HER2 status | EC^a^, EpCAM | Consistency, EpCAM | EC, HER2 | Consistency^b^, HER2 | Consistency^c^, HER2 |
| --- | --- | --- | --- | --- | --- | --- | --- | --- |
| 1 | 68 | Healthy | / | 360 | yes | 282 | yes | / |
| 2 | 61 | Healthy | / | 356 | yes | 316 | no | / |
| 3 | 30 | Healthy | / | 456 | no | 341 | yes | / |
| 4 | 49 | Healthy | / | 351 | yes | 195 | no | / |
| 5 | 60 | Healthy | / | 340 | yes | 269 | yes | / |
| 6 | 46 | Healthy | / | 437 | no | 241 | no | / |
| 7 | 59 | Healthy | / | 436 | no | 323 | no | / |
| 8 | 22 | Healthy | / | 407 | yes | 347 | yes | / |
| 9 | 68 | Healthy | / | 421 | yes | 375 | yes | / |
| 10 | 37 | Healthy | / | 329 | yes | 136 | no | / |
| 11 | 38 | Healthy | / | 352 | yes | 268 | no | / |
| 12 | 62 | Healthy | / | 335 | yes | 382 | no | / |
| 13 | 61 | Healthy | / | 365 | yes | 284 | yes | / |
| 14 | 36 | Healthy | / | 367 | yes | 321 | no | / |
| 15 | 59 | Benign | / | 357 | yes | 319 | no | / |
| 16 | 66 | Benign | / | 332 | yes | 298 | yes | / |
| 17 | 40 | Benign | / | 423 | yes | 312 | yes | / |
| 18 | 52 | Benign | / | 412 | yes | 256 | no | / |
| 19 | 29 | Benign | / | 367 | yes | 378 | no | / |
| 20 | 25 | Benign | / | 387 | yes | 334 | no | / |
| 21 | 30 | Benign | / | 426 | yes | 321 | no | / |
| 22 | 31 | Benign | / | 423 | yes | 356 | no | / |
| 23 | 36 | Malignant | negative | 784 | yes | 239 | yes | yes |
| 24 | 37 | Malignant | negative | 613 | yes | 346 | yes | yes |
| 25 | 52 | Malignant | negative | 579 | yes | 298 | yes | yes |
| 26 | 46 | Malignant | negative | 691 | yes | 375 | no | yes |
| 27 | 81 | Malignant | negative | 588 | yes | 284 | yes | yes |
| 28 | 47 | Malignant | negative | 428 | no | 378 | yes | yes |
| 29 | 72 | Malignant | negative | 568 | yes | 336 | yes | yes |
| 30 | 44 | Malignant | negative | 435 | no | 280 | yes | yes |
| 31 | 56 | Malignant | negative | 719 | yes | 257 | no | yes |
| 32 | 39 | Malignant | negative | 753 | yes | 388 | yes | yes |
| 33 | 28 | Malignant | negative | 633 | yes | 367 | yes | yes |
| 34 | 29 | Malignant | negative | 449 | yes | 365 | yes | yes |
| 35 | 83 | Malignant | negative | 664 | yes | 314 | yes | yes |
| 36 | 33 | Malignant | negative | 521 | yes | 361 | yes | yes |
| 37 | 63 | Malignant | negative | 638 | yes | 356 | yes | yes |
| 38 | 45 | Malignant | negative | 649 | yes | 268 | yes | yes |
| 39 | 62 | Malignant | negative | 466 | yes | 306 | yes | yes |
| 40 | 58 | Malignant | negative | 578 | yes | 348 | yes | yes |
| 41 | 53 | Malignant | negative | 422 | no | 350 | yes | yes |
| 42 | 32 | Malignant | negative | 615 | yes | 364 | yes | yes |
| 43 | 35 | Malignant | negative | 648 | yes | 336 | yes | yes |
| 44 | 48 | Malignant | negative | 794 | yes | 378 | yes | yes |
| 45 | 74 | Malignant | negative | 710 | yes | 375 | yes | yes |
| 46 | 61 | Malignant | negative | 685 | yes | 360 | yes | yes |
| 47 | 38 | Malignant | negative | 650 | yes | 434 | yes | no |
| 48 | 40 | Malignant | negative | 709 | yes | 313 | yes | yes |
| 49 | 75 | Malignant | negative | 596 | yes | 357 | yes | yes |
| 50 | 45 | Malignant | negative | 415 | no | 384 | yes | yes |
| 51 | 48 | Malignant | negative | 408 | no | 356 | yes | yes |
| 52 | 54 | Malignant | negative | 461 | yes | 407 | no | no |
| 53 | 76 | Malignant | negative | 737 | yes | 332 | yes | yes |
| 54 | 48 | Malignant | negative | 523 | yes | 335 | yes | yes |
| 55 | 64 | Malignant | negative | 719 | yes | 325 | yes | yes |
| 56 | 77 | Malignant | negative | 715 | yes | 423 | yes | no |
| 57 | 62 | Malignant | negative | 696 | yes | 378 | yes | yes |
| 58 | 46 | Malignant | negative | 716 | yes | 356 | yes | yes |
| 59 | 50 | Malignant | negative | 693 | yes | 378 | yes | yes |
| 60 | 32 | Malignant | negative | 754 | yes | 337 | yes | yes |
| 61 | 56 | Malignant | negative | 742 | yes | 366 | yes | yes |
| 62 | 44 | Malignant | negative | 555 | yes | 341 | yes | yes |
| 63 | 43 | Malignant | negative | 752 | yes | 432 | yes | no |
| 64 | 56 | Malignant | negative | 567 | yes | 432 | yes | no |
| 65 | 34 | Malignant | positive | 598 | yes | 494 | yes | yes |
| 66 | 46 | Malignant | positive | 653 | yes | 419 | yes | yes |
| 67 | 48 | Malignant | positive | 421 | no | 420 | yes | yes |
| 68 | 50 | Malignant | positive | 723 | yes | 405 | yes | yes |
| 69 | 52 | Malignant | positive | 567 | yes | 431 | no | yes |
| 70 | 40 | Malignant | positive | 552 | yes | 333 | yes | no |
| 71 | 45 | Malignant | positive | 598 | yes | 528 | yes | yes |
| 72 | 37 | Malignant | positive | 687 | yes | 450 | yes | yes |
| 73 | 75 | Malignant | positive | 732 | yes | 616 | yes | yes |
| 74 | 64 | Malignant | positive | 486 | yes | 332 | yes | no |
| 75 | 55 | Malignant | positive | 401 | no | 448 | no | yes |
| 76 | 69 | Malignant | positive | 523 | yes | 502 | yes | yes |
| 77 | 70 | Malignant | positive | 403 | no | 485 | yes | yes |
| 78 | 30 | Malignant | positive | 575 | yes | 456 | yes | yes |
| 79 | 39 | Malignant | positive | 555 | yes | 433 | yes | yes |
| 80 | 45 | Malignant | positive | 632 | yes | 502 | yes | yes |
| 81 | 76 | Malignant | positive | 403 | no | 468 | yes | yes |
| 82 | 46 | Malignant | positive | 449 | yes | 402 | yes | yes |
| 83 | 56 | Malignant | positive | 576 | yes | 523 | yes | yes |
| 84 | 65 | Malignant | positive | 587 | yes | 432 | yes | yes |
| 85 | 66 | Malignant | positive | 402 | no | 476 | yes | Yes |

^a^: Electrochemistry intensity. ^b^: Distinguishing breast cancer from healthy individuals. ^c^: Evaluating HER2 status in breast cancer patients.

**Table S18**. Comparison of emerging methods for isolation of sEVs.

| Methods | Isolation system | Time (min)^a^ | AUC^b^ | Verify practicality | Quantification | Reference |
| --- | --- | --- | --- | --- | --- | --- |
| Immunocapture | Antibody; screen-printed electrode; magnetic beads | 15 | 0.96 | Human plasma sample (n = 162) | Electrochemical | ^[49]^ |
| SEC^c^ | Filter membrane; ultrafilter; SEC column | < 20 | Not mentioned | Human serum samples (n = 3) | PEFD^d^ | ^[50]^ |
| SEC | Filter membrane; ultrafilter; antibody; streptavidin | 60 | Not mentioned | Human serum sample (n = 3) | Fluorescence | ^[51]^ |
| SEC | Filter membrane; ultrafilter; chromatography column; streptavidin; sepharose CL-4B | > 30 | 0.95 | Human serum sample (n = 51) | Fluorescence | ^[52]^ |
| Meso–macroporous hydrogel | PEG700DA meso–macroporous hydrogel | 80 | Not mentioned | Multiple biofluids | Multiple approaches | ^[53]^ |
| Precipitation | Aptamer; HCR^e^; sodium alginate; EDTA | 80 | 1.00 | Not mentioned | Fluorescence | ^[54]^ |
| Microfluidic Chip | Antibody; SiO_2_; PDMS^f^ chaotic mixer | > 150 | 0.88 | Human plasma sample (n = 30) | qPCR | ^[55]^ |
| Microfluidic Chip | Magnetic beads; liposome; aptamer; HB-Chip^g^; fluid supported lipid bilayer | 30 | 1.00 | Human plasma samples (n = 31) | Fluorescence | ^[56]^ |
| Microfluidic Chip | PDMS; GOQDs; antibody; Photothermal-driven | Not mentioned | Not mentioned | Breast cancer tissue (n = 3) | Fluorescence | ^[57]^ |
| Microfluidic Chip | Nano membrane; PMMA; centrifugation; HAPR^h^ | > 900 | 0.85 | whole blood (n = 22) | Colorimetric | ^[58]^ |
| Magnetic separation | Fe_3_O_4_; MXene; CD63 aptamer; polyethylenimine | 45 | Not mentioned | Not mentioned | Fluorescence | ^[59]^ |
| Magnetic separation | Magnetic nanomixers; polydopamine; antibody; Exonuclease I; SH-PEG | 60 | 1.00 | Human plasma sample (n = 30) | SERS^i^ | ^[41]^ |
| Affinity capture | ZrMOF; polyethylenimine; glass microbubbles | 90 | 1.00 | Human plasma sample (n = 55) | SERS | ^[60]^ |
| Affinity capture | Syringe filters; MXene; TiO₂; Fe₃O₄; | < 10 | 0.996 | Human urine sample (n = 225) | Fluorescence | ^[61]^ |
| Membrane filter | EXODUS (nanoporous membrane; cartridge; harmonic oscillator; outlet) | 20 | Not mentioned | Human urine samples (n = 113) | high-throughput analysis | ^[62]^ |
| Filter membrane | Filter membrane | < 10 | 0.965 | Human plasma sample (n = 85) | Electrochemical | This work |

^a^: Excluding exosome detection; ^b^: Area under the curve; ^c^: Size exclusion chromatography; ^d^: Plasmon-enhanced fluorescence detection; ^e^: Hybridization chain reaction; ^f^: Polydimethylsiloxane; ^g^: Herringbone microfluidic chip; ^h^: Highly abundant protein removal reagent; ^i^: Surface-Enhanced Raman Scattering; ^k^: Herringbone microfluidic chip.

**Table S19.** Internal cross-validation performance for breast cancer diagnosis and HER2-status discrimination.

| Purpose | Model | Cross-validated AUC | Sensitivity | Specificity | Accuracy |
| --- | --- | --- | --- | --- | --- |
| Diagnosis | EpCAM | 0.946 ± 0.003 | 80.1% ± 1.2% | 94.3% ± 3.2% | 83.8% ± 1.1% |
| Diagnosis | HER2 | 0.812 ± 0.005 | 78.8% ± 3.2% | 68.4% ± 0.9% | 76.1% ± 2.3% |
| Diagnosis | EpCAM + HER2 | 0.970 ± 0.003 | 87.1% ± 1.1% | 97% ± 4.5% | 89.7% ± 1.2% |
| Subtypes | HER2 | 0.897 ± 0.004 | 84.9% ± 2.1% | 88.1% ± 0.3% | 87.1% ± 0.7% |

**Statistical analysis.**

Data analysis was performed using IBM SPSS Statistics 23, and Origin 2021 software. In Figure 6 and 7, the statistical differences were tested by Mann-Whitney U-test. *p < 0.05 was considered statistically significant. **p < 0.01 and ***p < 0.001 were considered highly significant. In Figure 6 and 7, receiver operating characteristic (ROC) curves were constructed to assess the accuracy of cancer detection based on individual sEVs protein biomarker analysis or combined analysis. The optimal cutoff value is determined by maximizing the Youden index (sensitivity + specificity - 1).^[63]^

**The Calculation of Sensitivity and Specificity.** To assess the diagnostic performance, we determined the sensitivity and specificity. Sensitivity (true positive rate) represents the percentage of diseased patients correctly identified, whereas specificity (true negative rate) indicates the percentage of healthy individuals correctly classified. The calculations were performed as follows:

In this study, a positive result was defined by detection of either electrochemical signal above threshold. TP represent correctly identified breast cancer cases, while FN indicate missed diagnoses. TN reflect accurate classification of healthy controls, whereas FP denote healthy individuals misclassified as positive. Sensitivity and specificity were calculated via ROC curve analysis, with optimal cutoff determination using Youden's index. These performance metrics demonstrate sample size dependency, as reflected in their 95% confidence intervals.

**References**

[1]	aJ. Y. Luan, A. Seth, R. Gupta, Z. Y. Wang, P. Rathi, S. S. Cao, H. Gholami Derami, R. Tang, B. G. Xu, S. Achilefu, J. J. Morrissey, S. Singamaneni, *Nat Biomed Eng* **2020**, *4*, 518-530; bP. P. Chen, Y. M. Meng, T. Y. H. Liu, W. Peng, Y. Gao, Y. Q. He, R. L. Qu, C. Y. Zhang, W. Hu, B. W. Ying, *ACS Nano* **2023**, *17*, 6998-7006.

[2]	Y. Ma, X. H. Shi, Y. Ding, X. H. Zhang, J. Lu, D. P. Sun, *Chem. Eng. J.* **2024**, *483*, 149362.

[3]	Y. X. Zhang, Z. W. Cai, R. Zou, R. L. Wang, R. N. Tan, L. Wang, Y. X. Wu, H. P. He, Y. B. He, G. Chang, *ACS Sens* **2025**, *10*, 1187-1196.

[4]	X. Y. Meng, X. Zhao, W. J. Liu, Y. Wang, J. Chen, K. Chang, P. P. Chen, *Nano Today* **2025**, *65*, 102854.

[5] T. T. Wang, Y. Q. Ran, L. Shi, F. Q. Zhao, B. Z. Zeng, *Chem. Eng. J.* **2024**, *486*, 150314.

[6] Sweety, D. Kumar, *J. Colloid Interface Sci.* **2023**, *652*, 549-556.

[7]	Y. Zhang, X. F. Liu, N. Li, Y. Xu, Y. Ma, Z. Huang, H. B. Luo, C. J. Hou, D. Q. Huo, *Biosens. Bioelectron.* **2023**, *230*, 115263.

[8] C.-Y. Huang, F.-Y. Lin, C.-J. Chang, C.-H. Lu, J.-K. Chen, *Anal. Chem.* **2023**, *95*, 986-993.

[9] W. Ma, M. Z. Sun, P. Fu, S. Li, L. G. Xu, H. Kuang, C. L. Xu, *Adv. Mater.* **2017**, *29*, 1703410.

[10]	Z. L. Hu, Z. L. Yang, M. J. Chen, W. Chen, W. J. Ma, J. Lu, D. P. Sun, *Biosens. Bioelectron.* **2024**, *266*, 116717.

[11] A. Balzerova, A. Opletalova, V. Ranc, R. Zboril, *Appl. Mater. Today* **2018**, *13*, 166-173.

[12]	X.-W. Zhang, M.-X. Liu, M.-Q. He, S. Chen, Y.-L. Yu, J.-H. Wang, *Anal. Chem.* **2021**, *93*, 6437-6445.

[13]	Z. H. Zhang, R. H. Hu, D. Fu, W. J. Liu, J. Qu, Y. Wang, X. H. Zeng, X. Zhao, X. Y. Meng, B. X. Qiu, X. Jiang, B. F. Liu, J. Chen, P. P. Chen, *Chem. Eng. J.* **2025**, *517*, 164315.

[14]	Q. Z. Liang, Q. X. Zhou, H. Shi, S. Wu, J. H. Yang, T. Y. Zeng, J. Zhao, Y. M. Yin, G. X. Li, *Nano Today* **2024**, *54*, 102143.

[15]	L. Y. Ye, X. X. Xu, A. H. Qu, L. Q. Liu, C. L. Xu, H. Kuang, *Nano Res.* **2024**, *17*, 5452-5460.

[16]	J. J. Xie, Z. D. Mu, M. Qing, J. Zhou, S. C. Sun, L. J. Bai, *Chem. Eng. J.* **2022**, *450*, 138362.

[17]	R. J. Zeng, Y. X. Li, Y. L. Li, Q. Wan, Z. S. Huang, Z. L. Qiu, D. P. Tang, *Research* **2022**, *2022*, 9831521.

[18]	Y. Zhang, N. Li, Y. Xu, N. Qi, L. Peng, M. Yang, C. J. Hou, D. Q. Huo, *Anal. Chem.* **2023**, *95*, 6586-6594.

[19]	W. Q. Wang, R. Han, M. Chen, X. L. Luo, *Anal. Chem.* **2021**, *93*, 7355-7361.

[20]	W. J. Liu, Y. Wang, P. J. Jiang, K. Huang, H. Zhang, J. Chen, P. P. Chen, *Biosens. Bioelectron.* **2024**, *261*, 116493.

[21]	Y. S. Wang, R. J. Zeng, S. Tian, S. Y. Chen, Z. L. Bi, D. P. Tang, D. Knopp, *Anal. Chem.* **2024**, *96*, 13663-13671.

[22] M. G. Yin, T. Wang, Z. J. Wang, L. N. Lu, B. W. Ding, J. D. Chi, X. R. Wang, L. Xue, F. J. Dai, B. X. Hu, J. Jiao, Q. Chen, *Biosens. Bioelectron.* **2025**, *288*, 117813.

[23]	C. H. Zhai, J. Long, J. He, Y. Zheng, B. Q. Wang, J. Y. Xu, Y. T. Yang, L. Jiang, H. Yu, X. T. Ding, *ACS Nano* **2023**, *17*, 16656-16667.

[24]	L.-L. Wu, Z.-L. Zhang, M. Tang, D.-L. Zhu, X.-J. Dong, J. Hu, C.-B. Qi, H.-W. Tang, D.-W. Pang, *Angew. Chem. Int. Ed.* **2020**, *59*, 11240-11244.

[25]	X. J. Qin, B. Q. Wei, Y. H. Xiang, H. Lu, F. F. Liu, X. C. Li, F. Yang, *Biosens. Bioelectron.* **2024**, *245*, 115828.

[26]	C. C. Liu, H. X. Lin, J. Y. Guo, C. Yang, J. Chen, W. L. Pan, B. B. Cui, J. J. Feng, Y. Zhang, B. Li, S. H. Yao, L. Zheng, *Chem. Eng. J.* **2023**, *471*, 144364.

[27]	T. Arab, E. R. Mallick, Y. Huang, L. Dong, Z. H. Liao, Z. Z. Zhao, O. Gololobova, B. Smith, N. J. Haughey, K. J. Pienta, B. S. Slusher, P. M. Tarwater, J. P. Tosar, A. M. Zivkovic, W. N. Vreeland, M. E. Paulaitis, K. W. Witwer, *J Extracell Vesicles* **2021**, *10*, e12079.

[28]	J. Li, Y. Y. Li, Q. Li, L. Sun, Q. Q. Tan, L. Y. Zheng, Y. Lu, J. Q. Zhu, F. L. Qu, W. H. Tan, *Angew. Chem. Int. Ed.* **2024**, *63*, e202314262.

[29]	P. Zhang, X. Zhou, M. He, Y. Q. Shang, A. L. Tetlow, A. K. Godwin, Y. Zeng, *Nat Biomed Eng* **2019**, *3*, 438-451.

[30]	Z. J. Yang, Y. Atiyas, H. F. Shen, M. J. Siedlik, J. Y. Wu, K. Beard, G. Fonar, J. P. Dolle, D. H. Smith, J. H. Eberwine, D. F. Meaney, D. A. Issadore, *Nano Lett.* **2022**, *22*, 4315-4324.

[31]	X. Xu, Y. Y. Zhang, J. J. Liu, S. J. Wei, N. Li, X. T. Yao, M. X. Wang, X. H. Su, G. S. Jing, J. Q. Xu, Y. Liu, Y. Lu, J. Cheng, Y. C. Xu, *ACS Nano* **2024**, *19*, 1271-1285.

[32]	T. J. Ren, Y. Z. Zhang, Y. X. Tong, Q. Zhang, T. H. Wang, Y. Wang, C. G. Yang, Z. R. Xu, *Nat. Commun.* **2025**, *16*, 3391.

[33]	S. J. Pan, Y. Zhang, A. Natalia, C. Z. J. Lim, N. R. Y. Ho, B. Chowbay, T. P. Loh, J. K. C. Tam, H. L. Shao, *Nat. Nanotechnol.* **2021**, *16*, 734.

[34]	K. Jiao, G. Wang, T. Cheng, H. Zhou, Z. Wang, Y. X. Liu, Y. R. Chen, Y. Z. Ma, J. Jiao, Q. Zhao, *ACS Nano* **2026**, *20*, 18479-18490.

[35]	Y. Li, J. Q. Deng, Z. W. Han, C. Liu, F. Tian, R. Xu, D. Han, S. H. Zhang, J. S. Sun, *J. Am. Chem. Soc.* **2021**, *143*, 1290-1295.

[36]	J. Q. Deng, S. Zhao, K. Xie, C. Liu, C. G. Sheng, J. H. Li, B. Dai, S. Wan, L. L. Li, J. S. Sun, *Angew. Chem. Int. Ed.* **2025**, *64*, e202417165.

[37]	J. Q. Deng, S. Zhao, J. H. Li, Y. C. Cheng, C. Liu, Z. Liu, L. L. Li, F. Tian, B. Dai, J. S. Sun, *Angew. Chem. Int. Ed.* **2022**, *61*, e202207037.

[38]	Y. J. Liu, Y. P. Fan, X. Q. Li, G. Tian, B. Shen, M. H. Li, K. Su, X. H. Fu, M. X. Zhang, Y. H. Wang, X. Y. Li, X. M. Li, S. J. Ding, *Angew. Chem. Int. Ed.* **2025**, *64*, e202501804.

[39]	L. Y. Zou, X. F. Liu, Y. Zhou, W. J. Mei, Q. Wang, X. H. Yang, K. M. Wang, *Sens. Actuators, B* **2022**, *351*, 130893.

[40]	L.-L. Xu, M. Wang, Y.-K. Wang, Y.-J. Chen, Y.-X. Zhang, Y.-Q. Zhang, S.-B. Cheng, M. Xie, W.-H. Huang, *Small* **2025**, *21*, e202502763.

[41]	G. H. Zhang, Y. H. Wang, W. H. Zhou, Y. M. Lei, J. S. Lu, W. J. Yin, Z. Zhu, C. Y. Yang, P. Zhang, *Angew. Chem. Int. Ed.* **2023**, *62*, e202315113.

[42]	X. M. Su, X. Y. Liu, Y. C. Z. Xie, M. Y. Chen, C. Zheng, H. Zhong, M. Li, *ACS Nano* **2023**, *17*, 4077-4088.

[43]	J. Park, M. Feng, J. B. Yang, H. F. Shen, Z. Y. Qin, W. Guo, D. A. Issadore, *ACS Nano* **2025**, *19*, 17884-17899.

[44]	Y. Y. Zhang, X. J. Qin, Z. Y. Xu, W. F. Liu, H. Lu, Y. Yang, J. Yang, X. C. Li, Y. Q. Zhang, F. Yang, *ACS Nano* **2025**, *19*, 8093-8107.

[45]	D. G. Mathew, P. Beekman, S. G. Lemay, H. Zuilhof, S. Le Gac, W. G. van der Wiel, *Nano Lett.* **2020**, *20*, 820-828.

[46]	J. Park, J. S. Park, C.-H. Huang, A. Jo, K. Cook, R. Wang, H.-Y. Lin, J. Van Deun, H. Y. Li, J. H. Min, L. Wang, G. Yoon, B. S. Carter, L. Balaj, G.-S. Choi, C. M. Castro, R. Weissleder, H. Lee, *Nat Biomed Eng* **2021**, *5*, 678-689.

[47]	L. M. Yang, J. G. Zhang, J. Zhang, T. Hou, Q. Gao, X. J. Liu, F. Li, *Anal. Chem.* **2025**, *97*, 2176-2185.

[48]	X. J. Liu, X. Gao, L. M. Yang, Y. C. Zhao, F. Li, *Anal. Chem.* **2021**, *93*, 11792-11799.

[49]	J. Park, J. S. Park, C.-H. Huang, A. Jo, K. Cook, R. Wang, H.-Y. Lin, J. Van Deun, H. Y. Li, J. Min, L. Wang, G. Yoon, B. S. Carter, L. Balaj, G.-S. Choi, C. M. Castro, R. Weissleder, H. Lee, *Nat Biomed Eng* **2021**, *5*, 678-689.

[50]	K. S. Kapoor, K. Harris, K. A. Arian, L. Ma, B. Schueng Zancanela, K. A. Church, K. M. McAndrews, R. Kalluri, *Bioact Mater* **2024**, *40*, 683-695.

[51]	H.-Y. Liu, R. Kumar, C. T. Zhong, S. Gorji, L. Paniushkina, R. Masood, U. A. Wittel, H. Fuchs, I. Nazarenko, M. Hirtz, *Adv. Mater.* **2021**, *33*, 2008493.

[52]	A. Jo, A. Green, J. E. Medina, S. Iyer, A. W. Ohman, E. T. McCarthy, F. Reinhardt, T. Gerton, D. Demehin, R. Mishra, D. L. Kolin, H. Zheng, J. Cheon, C. P. Crum, R. A. Weinberg, B. R. Rueda, C. M. Castro, D. M. Dinulescu, H. Lee, *Adv Sci* **2023**, *10*, e202301930.

[53]	J. Kim, M. J. Kang, G. Han, S. Hyung, M. Kim, M. Jang, H. K. Lee, Y. Seo, K. C. Gil, C. Kim, S. Song, S. Jeong, S. Kim, M. S. Kim, J. S. Shim, S. G. Kang, Y. C. Lee, S. Chung, I.-J. Cho, T. S. Shim, K. H. Song, J. Min, H. Seong, K. Lee, J. Lee, C. Lee, H. N. Kim, H. Lee, S. H. Kim, J. Y. Kang, K. W. Bong, N. Choi, *Nat. Nanotechnol.* **2025**, *20*, 1678-1687.

[54]	H. Q. Wang, J. Y. Sun, Q. Q. Zou, B. Du, H. Liu, Y. N. Luan, X. Wang, X. H. Yang, Q. Wang, K. M. Wang, *ACS Sens* **2025**, *10*, 4136-4144.

[55]	H. Y. Kang, L. Qiu, Y. C. Li, X. C. Xu, R. J. Pei, T. Q. Yang, L. Z. Yang, X. B. Xu, N. Sun, *Adv. Healthcare Mater.* **2025**, *14*, e202500439.

[56]	Q. Niu, Y. Shu, Y. Q. Chen, Z. Huang, Z. X. Yao, X. F. Chen, F. H. Lin, J. Z. Feng, C. Huang, H. Wang, H. M. Ding, C. Y. Yang, L. L. Wu, *Angew. Chem. Int. Ed.* **2023**, *62*, e202215337.

[57]	C. Wang, Y. Zhang, J. B. Wang, Y. R. Han, Y. H. Wang, M. Y. Sun, Y. B. Liang, M. Huang, Y. Yu, H. L. Hu, H. Liu, L. Han, *Adv. Mater.* **2025**, *37*, e202411259.

[58]	X. D. Zhao, X. Liu, T. C. Chen, H. Xie, S. J. Li, Y. Zhang, H. W. Zhang, Y. L. Cao, W. Du, X. J. Feng, X. Liu, Y. W. Li, P. Chen, Q. B. Li, B.-F. Liu, *ACS Nano* **2025**, *19*, 8948-8965.

[59]	H. Y. Cui, T. F. Zheng, N. N. Qian, X. Q. Fu, A. J. Li, S. Xing, X.-F. Wang, *Small* **2024**, *20*, e202402434.

[60]	X. J. Qin, Y. H. Xiang, L. F. Mao, Y. Yang, B. Q. Wei, H. Lu, X. C. Li, Y. Q. Zhang, F. Yang, *ACS Nano* **2024**, *18*, 14569-14582.

[61]	H. L. Chen, Y. Qi, C. Y. Yang, Q. F. Tai, M. Zhang, X.-Z. Shen, C. H. Deng, J. M. Guo, S. Jiang, N. R. Sun, *ACS Nano* **2023**, *17*, 23924-23935.

[62]	Y. C. Chen, Q. F. Zhu, L. M. Cheng, Y. Wang, M. Li, Q. S. Yang, L. Hu, D. D. Lou, J. Y. Li, X. J. Dong, L. P. Lee, F. Liu, *Nat Methods* **2021**, *18*, 212-218.

[63]	W. B. Li, B. Wang, T. Z. Li, Y. W. Ma, H. Y. Jin, J. L. Zhao, Z. W. Xue, N. Su, Y. Y. He, J. Q. Shi, X. C. Liu, X. Y. Liu, T. Z. Wang, J. W. Wang, C. Li, C. Yan, Y. Ma, Q. C. Qi, X. Y. Wang, W. G. Li, B. Huang, D. H. Wang, X. L. Wang, Y. Qu, X. G. Li, C. Qiu, N. Yang, *NPJ Digital Med* **2026**, *9*, 184.
